# Distinguishing Neuromyelitis Optica Spectrum Disorders Subtypes: A Study on AQP4 and C3d Epitope Expression in Cytokine‐Primed Human Astrocytes

**DOI:** 10.1002/glia.24675

**Published:** 2025-01-27

**Authors:** Marlen Alisch, Franziska Foersterling, Dario Zocholl, Bakhrom Muinjonov, Patrick Schindler, Ankelien Duchnow, Carolin Otto, Klemens Ruprecht, Tanja Schmitz‐Hübsch, Sven Jarius, Friedemann Paul, Volker Siffrin

**Affiliations:** ^1^ Experimental and Clinical Research Center, Max‐Delbrück‐Center for Molecular Medicine and Charité–Universitätsmedizin Berlin Berlin Germany; ^2^ Institute for Biometry and Clinical Epidemiology, Charité – Universitätsmedizin Berlin Berlin Germany; ^3^ Department of Neurology Charité – Universitätsmedizin Berlin, Corporate Member of Freie Universität Berlin and Humboldt‐Universität Zu Berlin Berlin Germany; ^4^ Molecular Neuroimmunology Group, Department of Neurology University of Heidelberg Heidelberg Germany; ^5^ Cluster of Excellence NeuroCure Clinical Research Center, Charité–Universitätsmedizin Berlin Berlin Germany

**Keywords:** AQP4, astrocyte, C3/C3d, C5b‐9, EAAT2, IL‐6, MOGAD, NMOSD

## Abstract

Neuromyelitis optica spectrum disorders (NMOSD) are severe autoimmune conditions affecting the central nervous system. In a subset of cases, no autoantibodies are detectable with the currently used routine assays. This study aimed to determine whether the levels of expression of aquaporin‐4 (AQP4), excitatory amino acid transporter 2 (EAAT2), or complement C3/C3d and C5b‐9 in human astrocytes following incubation with patient sera under inflammatory conditions differ between the various NMOSD subtypes and whether such differences can help to identify autoantibody‐mediated cases of NMOSD. Levels of AQP4, EAAT2, complement C3/C3d and C5b‐9 epitope expression on human astrocytes pretreated with various cytokines were quantitatively analyzed via indirect immunofluorescence after exposure to sera from patients with AQP4‐IgG seropositive, MOG‐IgG seropositive, and AQP4/MOG‐IgG double seronegative NMOSD. Significant differences in AQP4 and C3d epitope expression were observed, with IL‐17A, IL‐10, and IL‐6 pre‐treatment notably influencing astrocytic responses. Using uniform manifold approximation and projection (UMAP), patients were classified into clusters corresponding to AQP4‐IgG seropositive, MOG‐IgG seropositive, or double seronegative NMOSD. These results demonstrate distinct astrocytic staining patterns across NMOSD subtypes, providing a potential diagnostic tool for distinguishing between autoantibody‐mediated astrocytopathy and other cases. These findings suggest specific pathogenic mechanisms linked to each NMOSD subtype, which may have implications for tailoring therapeutic strategies based on cytokine involvement and astrocyte reactivity.

## Main Points

- An astrocyte assay enhanced by IL‐6, IL‐17A, and IL‐10 distinguished NMOSD subtypes via AQP4 and C3d epitope expression.
- Clustering analyses revealed autoantibody‐specific patterns, highlighting diagnostic and therapeutic potential.

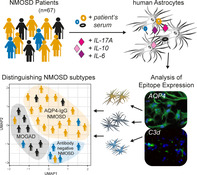

**Abbreviations**
ACM/ADM: astrocyte cultivation/differentiation medium
AF488/594: alexa fluor
AQP4: aquaporin‐4
ATP: adenosine triphosphate
AUC: area under the curve
BBB: blood–brain barrier (BBB)
bFGF: basic fibroblast growth factor
CBA: cell‐based assays
CNS: central nervous system
CNTF: ciliary neutrophic factor
CSF: cerebrospinal fluid
C3/C3d, C5b‐9, C1q, C3a: complement component 3/3d, 5b‐9
DAPI: 4′,6‐diamidino‐2‐phenylindole
EAAT2: excitatory amino acid transporter 2
EGF: epidermal growth factor
ELISA: enzyme‐linked immunosorbent assay
FBS: fetal bovine serum
HC: healthy controls
HEK: human embryonic kidney
hNSC: human neural stem cells
IF: immunofluorescence staining
IgG: immunoglobulin G
IL‐17A/‐10/‐6: interleukin ‐17A/‐10/‐6
IPND: International Panel for NMO Diagnosis
IVIG: intravenous immunoglobulins
IFNg: interferon‐gamma
MOG: myelin oligodendrocyte glycoprotein
MOGAD: MOG antibody disease
MS: multiple sclerosis
NMOSD: neuromyelitis optica spectrum disorders
PBS: phosphate‐buffered saline
ROC: receiver operating curve
RT: room temperature
Th17, Th1: T helper 17/1 cells
TNFa: tumor necrosis factor alpha
UMAP: Uniform Manifold Approximation and Projection

## 1 Introduction

Neuromyelitis optica spectrum disorders (NMOSD) encompass a group of severe inflammatory autoimmune diseases of the central nervous system (CNS) that are characterized by longitudinally extensive transverse myelitis, optic neuritis, brainstem encephalitis, and less commonly encephalopathy (Wingerchuk et al. 2015; Carnero Contentti and Correale 2021). The majority of patients with NMOSD have serum immunoglobulin G (IgG) autoantibodies against the water channel protein AQP4 (AQP4‐IgG) expressed by astrocytes (Jarius and Wildemann 2013; Jarius et al. 2020a). AQP4‐IgG serves as a biomarker that distinguishes NMOSD from multiple sclerosis (MS) and other neuroinflammatory disorders (Lennon et al. 2004, 2005; Jarius et al. 2020a). Different methods are used to detect AQP4‐IgG in patient sera, including live‐ or fixed cell‐based assays (CBA), tissue‐based assays, immunoprecipitation assays, and enzyme‐linked immunosorbent assay (ELISA) (Waters et al. 2014). This partly explains the divergent proportions of AQP4‐IgG seropositive patients reported in NMOSD cohorts. Live and fixed CBAs are the most accurate test for detecting AQP4‐IgG, with highest sensitivity and specificity (Waters et al. 2014, 2016; Redenbaugh et al. 2021). Approximately 10% of patients diagnosed with NMOSD according to the 2015 NMOSD criteria (Wingerchuk et al. 2015) do not have detectable serum levels of AQP4‐IgG. Among these AQP4‐IgG seronegative patients, about 30% exhibit antibodies targeting myelin oligodendrocyte glycoprotein (MOG), which is mainly expressed in oligodendrocytes (Mader et al. 2011; Jarius et al. 2018; Höftberger et al. 2020; Schanda et al. 2021). Recent findings support the assumption that IgG antibodies against MOG (MOG‐IgG) are a biomarker that defines a pathogenetically different disease with distinct clinical features, responses to treatment, and prognosis, termed MOG antibody‐associated disease (MOGAD) (Jarius et al. 2016, 2020b; a; Pache et al. 2016; Marignier et al. 2021; Banwell et al. 2023). However, there is overlap in some clinical and radiological features between AQP4‐IgG seropositive NMOSD and MOG‐IgG seropositive NMOSD/MOGAD (Sato et al. 2014; Reindl and Waters 2019). The diagnosis of AQP4‐/MOG‐IgG double seronegative NMOSD is currently made according to criteria proposed by the International Panel for NMO Diagnosis (IPND), which are based on clinical and radiological similarities with the classic phenotype associated with AQP4‐IgG seropositive NMSOD. However, AQP4/MOG‐IgG double seronegative NMOSD still poses a diagnostic and therapeutic challenges in clinical practice and its pathogenesis needs still to be elucidated (Yeo et al. 2019).

Binding of AQP4‐IgG to AQP4 on astrocytes has been found to be associated with complement activation via C1q and the expression of C3a in astrocytes leading to a loss of function of astrocytes (Hinson et al. 2008; Howe et al. 2014; Chen et al. 2020). Therefore, the downstream complement components C3 (opsonization) and C5 (induction of the membrane attack complex) are of specific interest in this context. Identification of the epitope has been shown to be an important feature of reactive astrocytes of the so‐called A1 phenotype, which exhibited neurotoxic properties (Liddelow et al. 2017).

Infiltration of immune cells and demyelination as well as severe neuronal injury have been described in NMOSD lesions (Bennett 2019; Jarius et al. 2020a). Consequently, binding of AQP4‐IgG to its target antigen is considered to be a crucial event in the immunopathogenesis of NMOSD (Crane et al. 2011). However, it remains unclear why AQP4‐IgG titers as measured by CBA do not consistently correlate with disease activity or treatment status (Jitprapaikulsan et al. 2020). One important factor in this context may be that the CBA employs immortalized cell lines, e.g., human embryonic kidney 293 (HEK293) transfected with human AQP4, in which there is transgenic and forced expression of AQP4, which does not recapitulate the physiologic expression levels on astrocytes. Interestingly, experimental data with astrocytes have shown a downregulation of the excitatory amino acid transporter 2 (EAAT2) (Hinson et al. 2008), following binding of AQP4‐IgG to astrocytes. EAAT2 is a major transporter protein clearing glutamate from the extracellular space at synapses in the central nervous system and is expressed predominantly in astrocytes. This indicates that AQP4 antibody binding has implications beyond cell lysis for astrocytes.

The cellular immunity of the disease has recently been in the focus of research efforts. Immune cell infiltrates in NMOSD forms have been shown to associate with certain cytokine expression patterns. Elevated levels of IL‐6 and IL‐17A were found in the CSF and serum of patients diagnosed with NMOSD (Uzawa et al. 2010; Li et al. 2011; Matsushita et al. 2013; Chang et al. 2015). IL‐1b has been shown to promote neuronal damage via the activation of microglia and astrocytes leading to the downstream synthesis of other pro‐inflammatory and chemotactic mediators within the CNS (Allan and Rothwell 2001; Shaftel, Griffin, and O'Banion 2008). TNFa secreted by activated monocytes and astrocytes acts on neighboring cells such as endothelial cells and leukocytes by promoting the recruitment of immune cells to the site of inflammation, inducing blood–brain barrier disruption, and triggering oxidative stress‐induced damage (Kwon and Koh 2020; Versele et al. 2022). IL‐10 acts as an anti‐inflammatory cytokine inhibiting neuroinflammation and promoting neuroprotection and repair by inhibiting the production of pro‐inflammatory cytokines and promoting the differentiation of regulatory T cells (Porro, Cianciulli, and Panaro 2020). Inhibition of the activity of IL‐6 has been found to be a highly efficient treatment strategy in AQP4‐IgG seropositive NMOSD to reduce inflammation and mitigate neural damage (Fujihara et al. 2020).

Based on the hypothesis that inflammatory cytokines are the main drivers of disease activity alongside the presence of autoantibodies, we developed a serological assay using cytokine‐pre‐incubated human astrocytes. We investigated the effect of sera from a large cohort of patients with NMOSD on the integrity of cytokine‐pre‐treated human astrocytes. Our study focused on the impact of sera from patients with NMOSD (AQP4‐IgG seropositive, MOG/AQP4‐IgG double seronegative, or MOG‐IgG seropositive) on the expression of AQP4, EAAT2, C3d, and C5b‐9 epitopes. Our objective was to determine if NMOSD subtypes could be classified by analyzing differences in the expression of these markers under different cytokine pre‐treatments with the ultimate goal of identifying autoantibody‐mediated astrocytopathies among all patients with NMOSD (Takeshita et al. 2021).

## 2 Methods

### 2.1 Serum Collection

**FIGURE 1:**
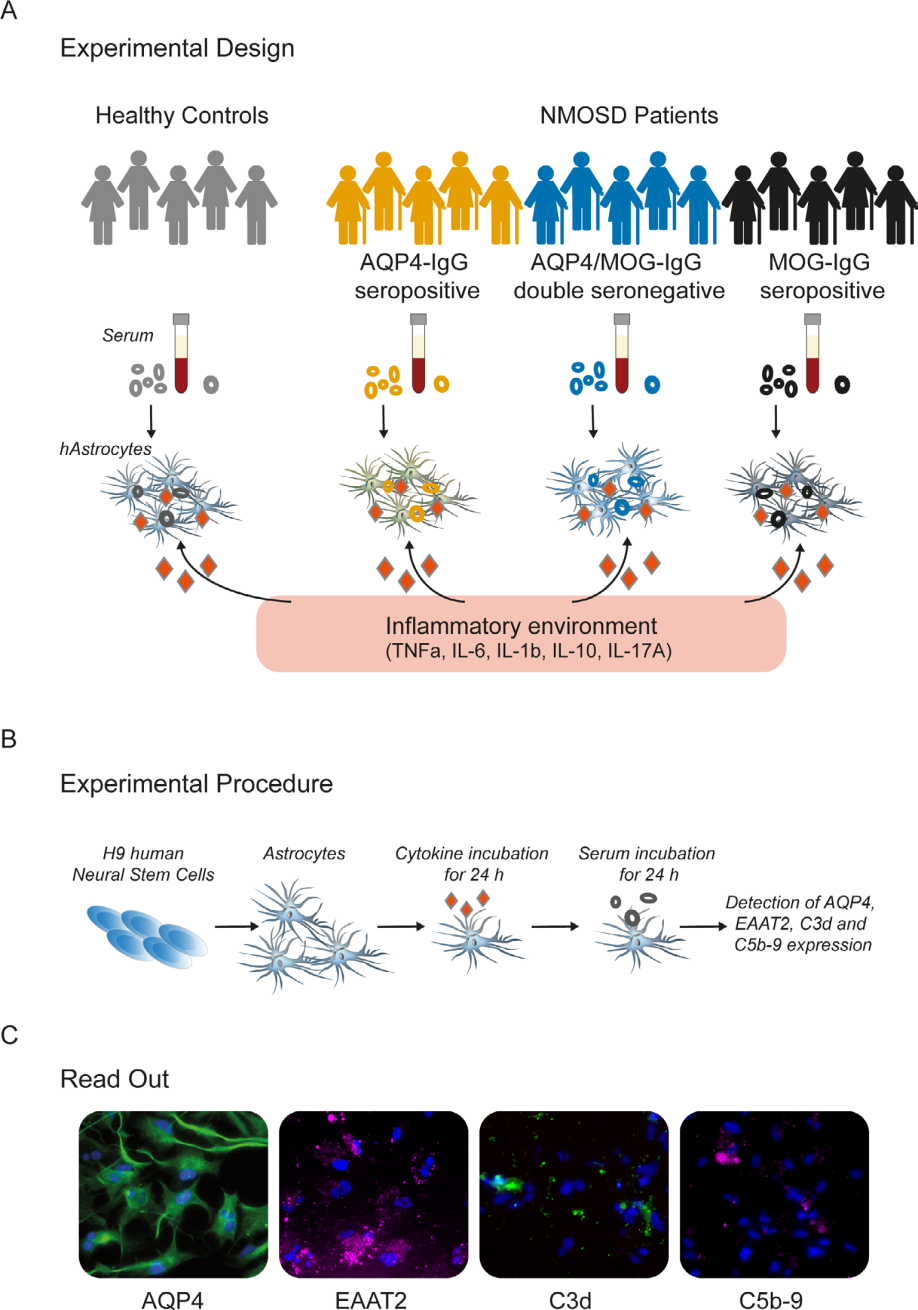
Experimental design, procedure, and readout. (A) Human astrocytes were exposed to serum from different NMOSD patient groups consisting of patients tested positive or negative for AQP4‐IgG and tested positive for MOG‐IgG in an inflammatory environment. Sex‐ and age‐matched HC were used as healthy controls. (B) First, human astrocytes were differentiated from H9‐derived neuronal stem cells and incubated with TNFa, IL‐6, IL1‐b, IL‐10 or IL‐17A for 24 h. After cytokine incubation, astrocytes were exposed to 10% serum from the different NMOSD patient groups. (C) For studying functional integrity of astrocytes, immunofluorescence stainings for aquaporin 4 (AQP4), excitatory amino acid transporter 2 (EAAT2), complement component 3d (C3d), free cleaved and within the C3 complex, and complement component C9 within the C5b‐9 complex (C5b‐9) were performed and images were analyzed.

Unselected sera of patients with NMOSD (*n* = 67) in a stable, non‐relapse phase and of healthy controls (HC) (*n* = 48) from the BERLimmun cohort study (Sperber et al. 2022) conducted at the NeuroCure Clinical Research Center and the Experimental and Clinical Research Center, Charité‐Universitätsmedizin, Germany, were included. The majority of patients received rituximab (*n* = 28), while a proportion received azathioprine therapy (*n* = 9) and a small number of patients received other treatments (mycophenolate‐mofetile *n* = 3, tocilizumab *n* = 1, methotrexate *n* = 1, intravenous immunoglobulins *n* = 1) during sampling. Twenty‐four patients were untreated. Approval for the study was granted by the Ethics Committee of Charité—Universitätsmedizin Berlin, and the study was conducted in adherence to the principles stated in the Helsinki declaration. All participants provided written informed consent. Patients included in the study have been diagnosed with AQP4‐IgG seropositive NMOSD based on the consensus criteria of 2015 or with MOGAD according to the criteria of Jarius et al. (Wingerchuk et al. 2015; Jarius et al. 2018). The titers of AQP4‐IgG and MOG‐IgG in the sera were determined by CBA using HEK293 cells overexpressing AQP4 or MOG (Jarius et al. 2010, 2020b). Patients were categorized as NMOSD‐AQP4‐IgG seropositive or NMOSD‐MOG‐IgG seropositive if they tested positive for one of these antibodies at least once during their disease course. Patients who tested negative for either autoantibody, and who met the 2015 consensus criteria for seronegative NMOSD, were regarded as NMOSD‐AQP4/MOG‐IgG double seronegative. Demographic data and clinical data are summarized in Figures 1 and S1.

### 2.2 Cell Culture

Human neural stem cells (hNSC) obtained from ThermoFisher Scientific (Schwerte, Germany) were cultivated according to the manufacturer's recommendations. Cells were maintained in an NSC medium, consisting of KnockOut D‐MEM/F12 (Gibco‐ThermoFisher Scientific) supplemented with 2 mM Glutamax (Gibco‐ThermoFisher Scientific), 2% StemPro Neural Supplement (Gibco‐ThermoFisher Scientific), 1% Penicillin–Streptomycin (Gibco‐ThermoFisher Scientific), 20 ng/μL bFGF (Peprotech, Hamburg, Germany), and 20 ng/μL EGF (Peprotech) in a humidified incubator at 37°C and with 5% CO_2_. Differentiation of NSC to astrocytes was performed according to a protocol established in our lab (Alisch et al. 2021; Kerkering et al. 2023). In short, for the differentiation of hNSCs to astrocytes, hNSCs were plated on Geltrex‐coated (ThermoFisher Scientific) 6‐well plates and cultivated in astrocyte differentiation medium (ADM), which contains D‐MEM (Gibco‐Thermo Fisher Scientific) supplemented with 2 mM Glutamax, 1% N‐2 (Thermo Fisher Scientific), 1% Penicillin–Streptomycin, 1% FCS (Sigma‐Aldrich‐Merck, Darmstadt, Germany), and 20 ng/mL CNTF (Miltenyi Biotec, Bergisch Gladbach, Germany). Cells were cultivated for 4 weeks, and medium was changed every 3–4 days. For experiments, astrocytes were plated on Geltrex‐coated 16‐well chamber slides in a density of 30,000 cells/0.5 cm^3^ and maintained in astrocyte cultivation medium (ACM) containing ADM without FCS for 5 days before starting experiments.

### 2.3 Cytokine Pre‐Treatment and Serum Exposure

First, astrocytes were incubated with the cytokines TNFa, IL‐1b, IL‐6, IL‐10, and IL‐17A (all from Peprotech), each in increasing concentrations of 0.1, 1, 10, and 50 ng/mL. After 24 h, medium was removed and replaced by ACM with 10% serum of 5 AQP4‐IgG‐positive NMOSD patients or healthy controls. After incubation with serum for 24 h, cells were used for immunofluorescence staining and viability assay (Figure 1). For all further experiments, pre‐treatment of 0.1 ng/mL TNFa, 0.1 ng/mL IL‐1b, 1 ng/mL IL‐6, 10 ng/mL IL‐10, and 10 ng/mL IL‐17 and serum samples (from patients with AQP4‐IgG seropositive, MOG‐IgG seropositive, or AQP4/MOG‐IgG double seronegative NMOSD or healthy controls) diluted 1:10 in ACM were used.

### 2.4 Immunofluorescence Staining

We performed immunofluorescence staining (IF) for AQP4, EAAT2, C3d, and C5b‐9 epitopes followed by fluorescence microscopy image analysis. For IF, astrocytes were seeded on Geltrex‐coated 16‐well chamber slides. After the incubation of astrocytes with cytokines followed by the exposure to patient's serum, cells were fixed by incubating them with 3.7% paraformaldehyde (Santa Cruz Biotechnology, Heidelberg, Germany) in phosphate‐buffered saline (PBS; Thermo Fischer Scientific) for 10 min at room temperature (RT). Then, cells were permeabilized using 0.1% Triton X‐100 (Sigma‐Aldrich‐Merck) for 5 min at RT. For staining, astrocytes were incubated with antibodies against AQP4 (dilution 1:100, E‐AB‐64864, Elabscience‐Biomol Hamburg, Germany), EAAT2 (dilution 1:250, ab41621, Abcam, Cambridge, UK), C3d (detecting an epitope in both cleaved C3d as well as in uncleaved C3 and C3b; dilution 1:200, A0063, Dako‐Agilent, Waldbronn, Germany) and C5b‐9 (dilution 1:200, M0777, Dako‐Agilent) by incubating for 1 h at RT. Following a washing step, cells were incubated with the corresponding secondary antibody AF488 goat anti‐mouse IgG (A11001), AF594 goat anti‐mouse IgG (A11032), AF488 donkey anti‐rabbit IgG (A21206), or AF594 goat anti‐rabbit IgG (A11012) each at a dilution of 1:1000 for 1 h at RT. All secondary antibodies were obtained from Invitrogen (Thermo Fisher Scientific). Cell nuclei were counterstained with DAPI (4′,6‐diamidino‐2‐phenylindole, Thermo Fisher Scientific) diluted 1:4000 and incubated for 10 min at RT. The chamber was removed from the slide and samples were mounted with fluorescence mounting medium (Dako‐Agilent). Images were obtained using an inverted Leica DMI6000B microscope and LAS X Life science software (Leica, Wetzlar, Germany).

### 2.5 Image Analysis

Image analyses were performed using the open‐source image processing software ImageJ, version 2.1.0/1.53c (http://imagej.net/Contributors). Ten randomly selected images of each experiment and condition were acquired blindly with a fluorescence microscope using same microscope settings (exposure time, gain, lamp intensity, magnification) for each experiment or test series with treatment and corresponding HC control. Every image consists of three single images, one for each channel: DAPI (UV channel), AQP4 or C3d epitope (green channel, Alexa 488), and EAAT2 or C5b‐9 (red channel, Alexa 594). For image analysis, images were split in their channels and each converted to a binary image. Using a verified threshold, the area of antigen staining and the signal intensity/integrated density were calculated. Area is defined as the number of square pixels; and signal intensity as the product of area and mean gray values. Further, cell nuclei were automatically counted by setting a verified threshold, defining the size of analyzed particles and counting the particles. Detected epitope area were related to the number of nuclei, allowing to examine the epitope areas per cell and quantify changes regarding expression of AQP4, EAAT2, and C3d and C5b‐9 epitopes.

For the morphometric analysis, images were processed using an algorithm written in FIJI17 image analysis software. All channels were split, and AQP4 stained images were analyzed separately to define the morphological parameters of individual cells. The channel with AQP4 staining was preprocessed with appropriate thresholding, filtered with “tubeness” (Sato et al. 1998) plugin for robust delineation of shape of elongated astrocytic processes. Subsequently masked images were analyzed to count the number, total area, and average size of detected particles.

### 2.6 Viability Assay

For the examination of the viability of astrocytes after incubation with inflammatory cytokines and patient sera, CellTiter‐Glo Luminescent Cell Viability Assays (Promega) were used according to the manufacturer's protocol. The test is based on the detection of ATP associated with the overall production energy and the metabolic activity in cells. For this purpose, astrocytes were lysed after incubation and the amount of ATP in astrocytes was detected by luciferase enzyme reactivity detection. Three technical replicates were measured for each individual experiment.

### 2.7 Statistics

Statistical analysis was performed with GraphPad Prism (version 8.3.0) and the open‐source software R version4.1 (R Core Team R 2021). Significant differences were determined using the non‐parametric Wilcoxon matched‐pairs signed rank test for paired data (HC vs. NNMOSD) and Mann–Whitney U Test for unpaired data (comparison of the NMOSD subgroups vs. HC). Statistical significance was defined as *p* < 0.05. Logistic regression was used to predict the probability of either case or control. Multiple measurements per individual were accounted for by a mixed effects regression with random intercept using the R package lme4 (Bates et al. 2015). Since in some instances, estimation methods based on maximum likelihood produced singular fits, Bayesian regression with default weakly informative priors was used as implemented in brms (Bürkner 2017). Using these probabilities as continuous predictor, receiver operating curve (ROC) curves and their area under the curve (AUC) were calculated using pROC (Robin et al. 2011). At multiple stages during the data collection, it was decided which assays should be continued based on the calculated statistical power for an AUC greater than 0.5 assuming this assay would be continued until the end of study. This power analysis was again performed with pROC (Robin et al. 2011).

UMAP analysis was performed using the UMAP package in R, applying the dimensionality reduction algorithm described by McInnes, Healy, and Melville (https://doi.org/10.48550/arXiv.1802.03426), with further details available in the package documentation (https://cran.r‐project.org/web/packages/umap/vignettes/umap.html). Only complete datasets were included in the analysis, defined as those containing data for AQP4 and C3d epitope expression levels after astrocyte incubation with NMOSD patient sera and exposure to every cytokine treatment (IL‐10, IL‐17, IL‐6, and control). Datasets were excluded if microscopy quality was inadequate (e.g., due to air bubbles or artifacts) or if there were significant differences in cell density between conditions, making those datasets unsuitable. For UMAP analyses, AQP4 and C3d epitope area expression values were normalized relative to the corresponding HC with the same cytokine treatment, ensuring comparability across experimental runs and between NMOSD subtypes.

## 3 Results

### 3.1 Receiver Operating Characteristic Analyses Were Used to Establish Optimal Cytokine Levels for Astrocyte Pre‐Conditioning

**FIGURE 2:**
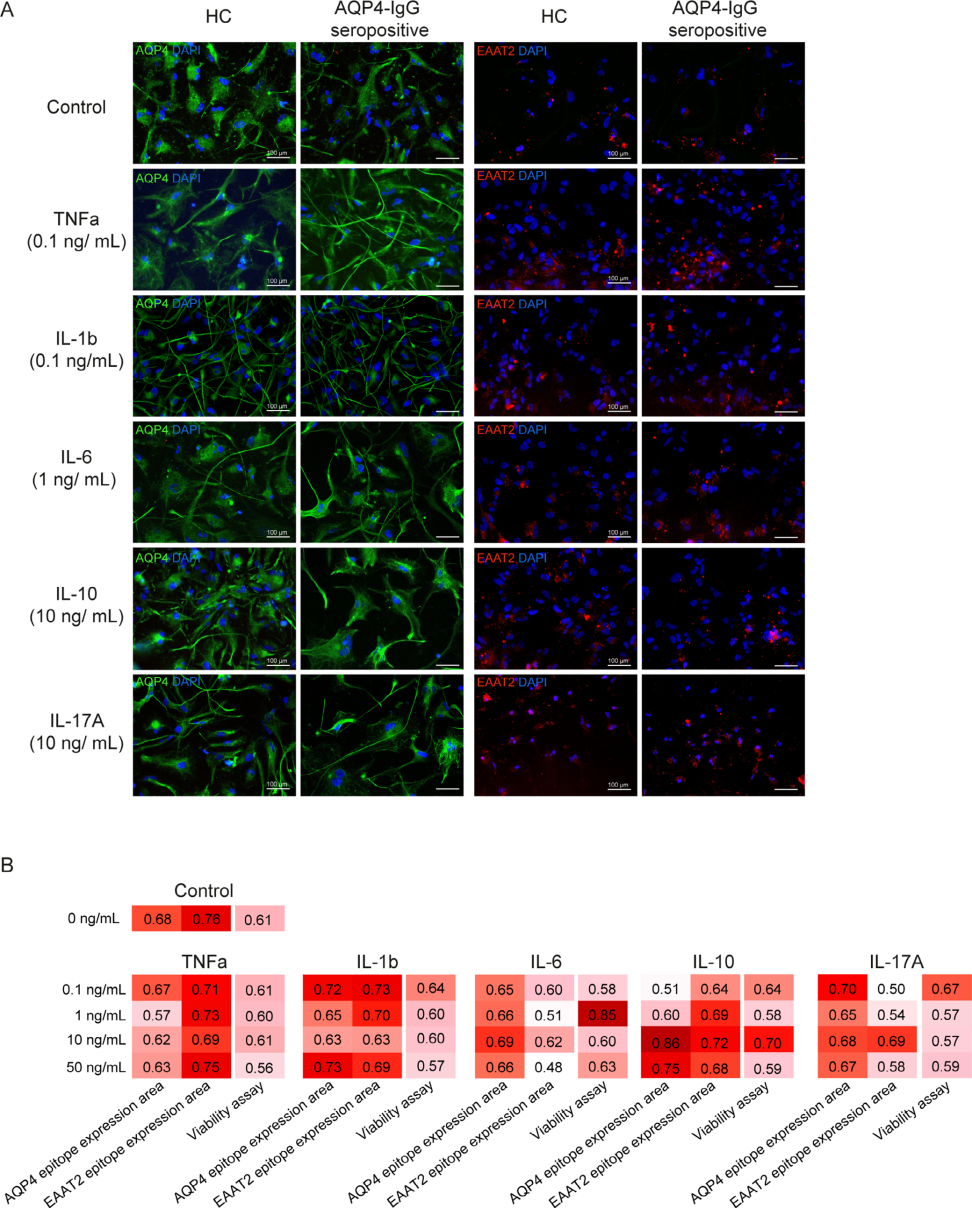
Results of cytokine titration experiments to determine the best pre‐incubation conditions. (A) Comparison of AQP4 and EAAT2 epitope expression in astrocytes pre‐incubated with 0.1 ng/mL TNFa, IL‐1b, 1 ng/mL IL‐6 and 10 ng/mL IL‐10 or IL‐17A and exposed to 10% sera from AQP4‐IgG seropositive NMOSD patients and healthy individuals. (B) ROC curve results of cytokine titration (0.1, 1, 10, and 50 ng/mL TNFa, IL‐1b, IL‐6, IL‐10, and IL‐17A) in combination with exposure to sera from AQP4‐IgG seropositive NMOSD patients (*n* = 5). Based on the results of AQP4 and EAAT2 epitope expression area and of a performed viability assay, AUC for experimental level classification were determined. Best results were achieved when astrocytes were incubated with 0.1 ng/mL TNFa, IL‐1b, 1 ng/mL IL‐6 and 10 ng/mL IL‐10 and IL‐17A.

In the initial, dose‐finding set of experiment serum from 5 patients with AQP4‐IgG seropositive NMOSD and from 5 HC were used. AQP4 and EAAT2 expression was analyzed by IF (Figures 2A, S2 and S3), and image analysis conducted by measuring area of antigen expression and signal intensity (Figure 2A). We evaluated metabolic activity and integrity through a viability assay based on ATP quantification in lysed astrocytes, which showed no major toxicity of any of the investigated conditions. Interestingly, there were divergent trends of viability after cytokine treatment (IL‐6 increase, IL‐10/IL‐17A decrease, Figure S4).

To visualize and quantify the sensitivity and specificity of each test setting, receiver operating characteristic (ROC) curve analyses were performed for all IF data and the viability assay (Figure 2B). Based on these findings, we identified the highest area under the curve (AUC) values when astrocytes were pre‐incubated with 0.1 ng/mL TNFa, 0.1 ng/mL IL‐1b, 1 ng/mL IL‐6, 10 ng/mL IL‐10, and 10 ng/mL IL‐17A. We used these concentrations for all subsequent assays.

### 3.2 IL‐17A, IL‐10, or IL‐6 Pre‐Treatment Enable Distinction Between AQP4‐IgG Seropositive NMOSD and HC Sera by Analysis of AQP4 and C3d Epitope Expression

**FIGURE 3:**
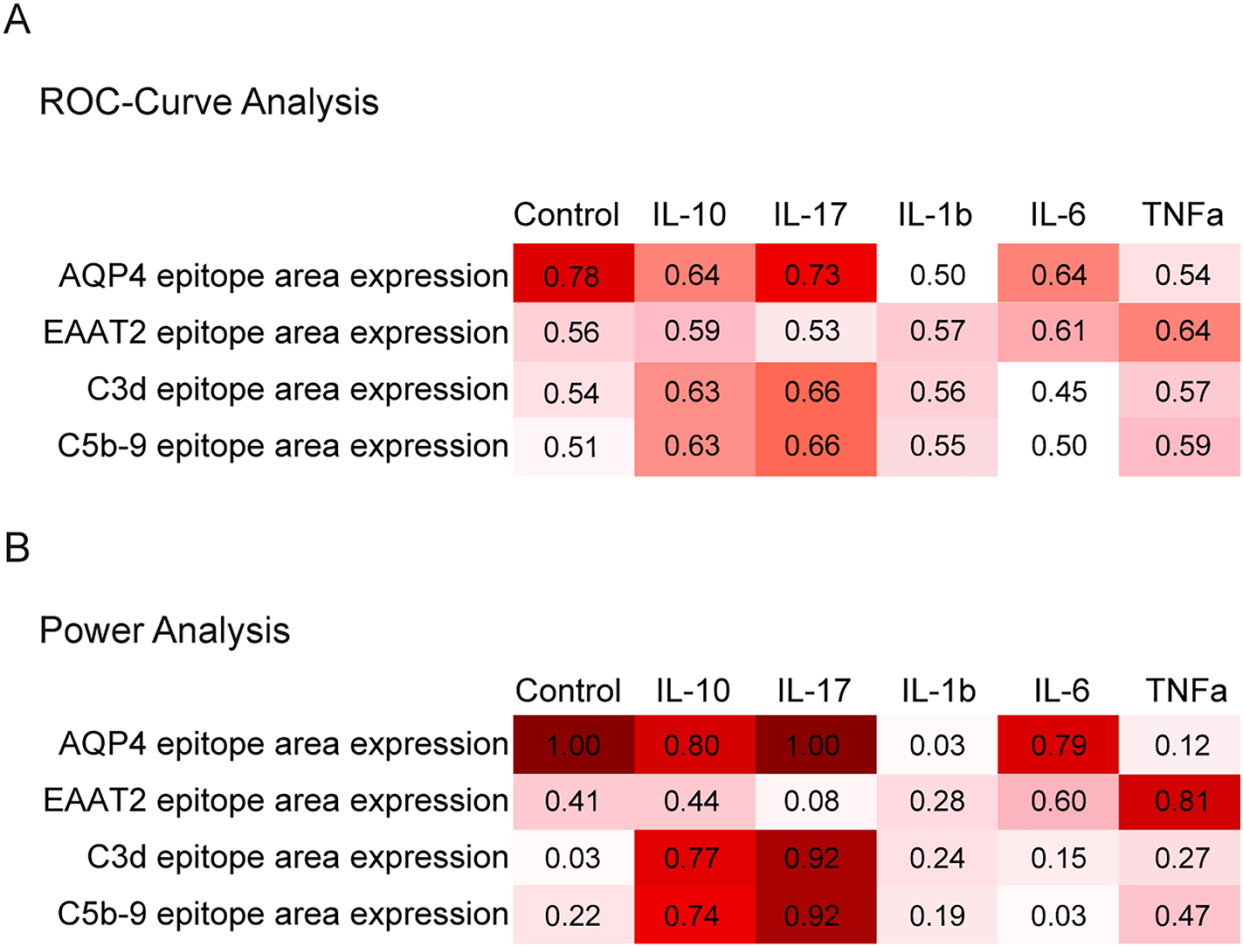
ROC curve and power analyses of different testes assay conditions. Astrocytes were pre‐incubated with TNFa (0.1 ng/mL), IL‐1b (0.1 ng/mL), IL‐6 (1 ng/mL), IL‐10 (10 ng/mL), and IL‐17A (10 ng/mL) and exposed to 10% sera from NMOSD‐AQP4‐IgG seropositive patients and HC (*n* = 16–18). Data are evaluated for AQP4, EAAT2, C3d, and C5b‐9 antigen area expression. (A) Results of determined AUC values of experimental level classification and (B) results of power analysis are shown. Best results were achieved for the analysis parameter AQP4 and C3d in IL‐10, IL‐17A, and IL‐6 pre‐treatment conditions.

Next, we confirmed the feasibility of cytokine pre‐conditioning settings with a larger set of AQP4‐IgG seropositive NMOSD sera. We analyzed epitope expression levels of AQP4, EAAT2, C3d, and C5b‐9. ROC curve analysis identified the respective analysis parameters with the best results for each cytokine pre‐treatment (Figure 3A). Power analyses were used to evaluate parameters that could be reliably used for analysis in the total cohort (*n* = 67) (Figure 3B). Calculated AUC values and power values were highest for AQP4 and C3d epitope expression/signal intensity as analytes (AQP4: AUC^highest^ = 0.78, Power^highest^ = 1.0; C3d: AUC^highest^ = 0.72, Power^highest^ = 1.0). EAAT2 or C5b‐9 assay resulted in lower AUC and power values (EAAT2: AUC^highest^ = 0.64, Power^highest^ = 0.81; C5b‐9: AUC^highest^ = 0.66, Power^highest^ = 0.92). Distinct antigen expression results regarding cytokine pre‐treatment were achieved when astrocytes were pre‐incubated with IL‐10 (AQP4: AUC = 0.64 Power = 0.8; C3: AUC = 0.63, Power = 0.77), IL‐17A (AQP4: AUC = 0.73, Power = 1.0; C3d: AUC = 0.66, Power = 0.92), IL‐6 (AQP4: AUC = 0.64, Power = 0.79) or without pre‐treatment (AQP4: AUC = 0.78, Power = 1.0). Based on these results, we chose untreated, IL‐17A, IL‐10, and IL‐6 as final pre‐conditioning. We focused our analysis on AQP4 and C3d epitopes as best parameters to analyze the whole cohort of sample pairs.

### 3.3 AQP4 Epitope Expression Is Differentially Affected in NMOSD Subcategories With and Without Cytokine Pre‐Conditioning

**FIGURE 4:**
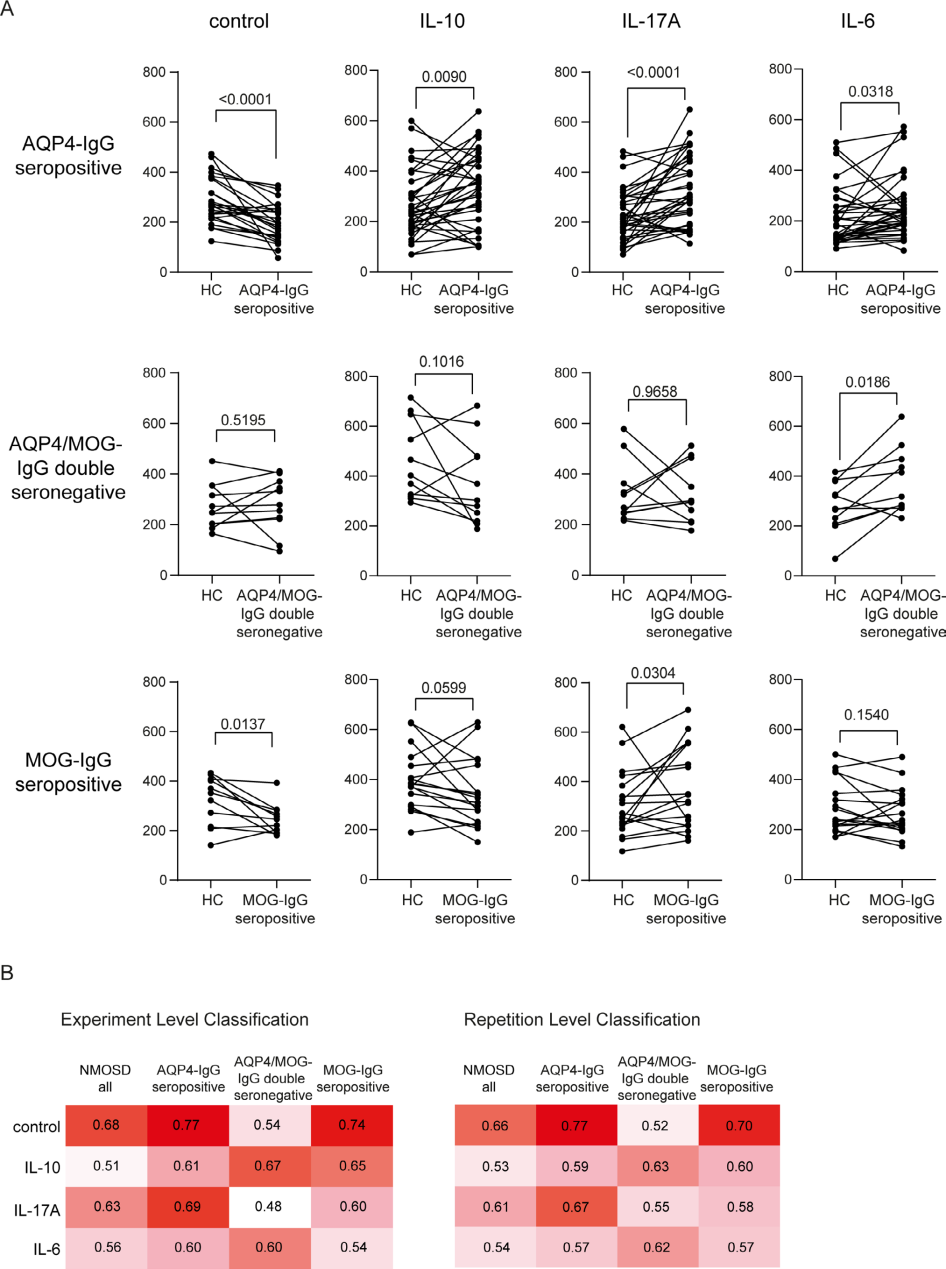
Determination of AQP4 epitope expression per nuclei in astrocytes incubated with IL‐10 (10 ng/mL), IL‐17 (10 ng/mL), IL‐6 (1 ng/mL) or without pretreatment and exposed to sera from NMOSD‐patients and from HC. (A) Single values of every experiment and statistical evaluation are depicted for NMOSD‐AQP4‐IgG+ (*n* = 25–36), for NMOSD‐MOG/AQP4‐IgG‐negative (*n* = 10–11), and for NMOSD‐MOG‐IgG+ (*n* = 11–18) Statistical test: Wilcoxon matched‐pairs signed rank test, *p*‐values are presented with significance determined at thresholds of *p* < 0.05, *p* < 0.01, *p* < 0.001, and *p* < 0.0001. (B) Results of determined AUC values regarding experimental level and repetition level classification.

Our cohort consisted of 67 patients with NMOSD (38 AQP4‐IgG seropositive, 11 AQP4/MOG‐IgG double seronegative, 18 MOG‐IgG seropositive). First, we pair‐wise compared age‐ and sex‐matched HC with the three NMOSD subtypes (Figure 4A). Under control conditions, expression of AQP4 was significantly decreased after incubation with AQP4‐IgG seropositive sera (*p* < 0.0001) and MOG‐IgG seropositive sera (*p* < 0.0137) compared to HC sera. Pre‐treatment of astrocytes with IL‐17A, IL‐10, or IL‐6 and exposure to sera from NMOSD‐AQP4‐IgG seropositive NMOSD led to a significant increase of AQP4 expression (IL‐17: *p* < 0.0001, IL‐10: *p* < 0.0090, IL‐6: *p* < 0.0318) compared to HC. In the MOG‐IgG seropositive group, this effect was only observed after IL‐17A pre‐treatment (*p* < 0.0304). Significant effects from AQP4/MOG‐IgG double seronegative sera compared to HC sera could only be determined when astrocytes were pre‐incubated with IL‐6. Here, incubation with patient sera was associated with an increase in AQP4 expression (*p* < 0.0186) (Figure 4A). To illustrate the specific modification of astrocyte morphology upon cytokine pre‐treatments in context of serum incubation, we added representative IF images to the supplement (Figure S5) and a morphometric analysis of these data (Figure S6). Given that sex is a significant susceptibility factor for NMOSD, we also analyzed potential sex‐specific differences in the AQP4 expression assay. However, although the sample size for men was limited, we did not observe pervasive signals or trends indicating differential results across NMOSD subtypes (with one possible exception for MOG‐IgG positive NMOSD sera and IL‐6 preconditioned astrocytes; Figure S7).

To differentiate between the different NMOSD subtypes, we performed a ROC analysis and calculated AUCs for the whole cohort. The highest AUC values (experimental and repetition level classification) regarding AQP4 expression were observed after incubation of astrocytes with AQP4‐IgG seropositive sera for the control condition (0.77/0.77) and IL‐17A pre‐treatment (0.69/0.67); after exposure to AQP4/MOG‐IgG double seronegative sera after IL‐10 (0.67/0.63) and IL‐6 (0.6/0.62) pre‐treatment; and after exposure to MOG‐IgG seropositive sera under control conditions (0.74/0.70) (Figure 4B).

### 3.4 C3d Epitope Expression Patterns Are Distinct From AQP4 Expression Pattern in NMOSD Subcategories

**FIGURE 5:**
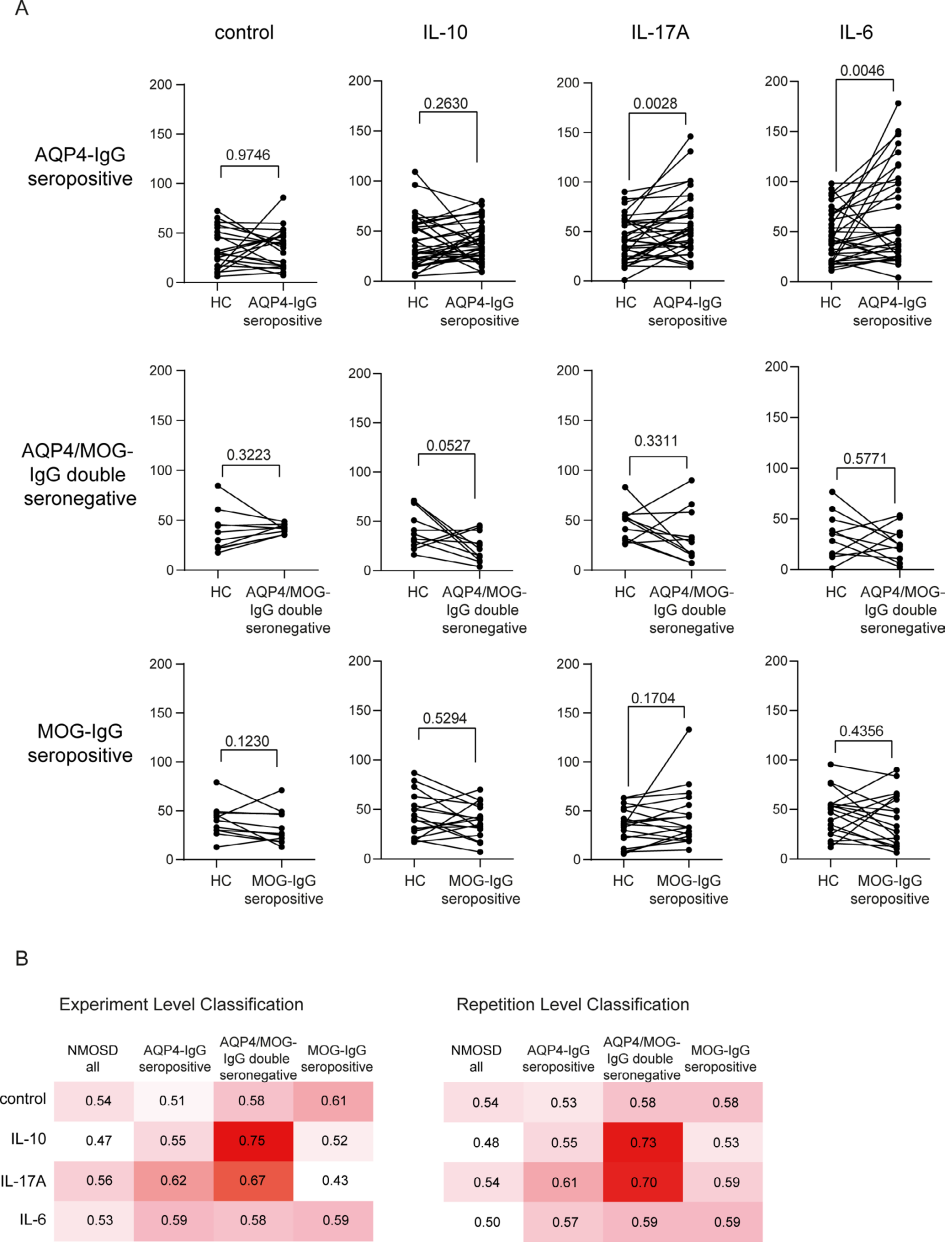
Determination of C3d epitope expression per nuclei in astrocytes incubated with IL‐10 (10 ng/mL), IL‐17 (10 ng/mL), IL‐6 (1 ng/mL) or without pretreatment and exposed to sera from NMOSD‐patients and from HC. (A) Single values of every experiment and statistical evaluation are depicted for NMOSD‐AQP4‐IgG seropositive (*n* = 25–36), for NMOSD‐MOG/AQP4‐IgG double seronegative (*n* = 10–11), and for NMOSD‐MOG‐IgG seropositive (*n* = 11–18) Statistical test: Wilcoxon matched‐pairs signed rank test, *p*‐values are presented with significance determined at thresholds of *p* < 0.05, *p* < 0.01, *p* < 0.001, and *p* < 0.0001. (B) Results of determined AUC values regarding experimental level and repetition level classification.

We analyzed C3d epitope expression in our NMOSD cohort of 67 individuals. Under the control treatment condition, no significant changes in C3d epitope expression were observed (Figure 5A). In IL‐17A and IL‐6 pre‐treated astrocytes, a significantly higher expression of C3d epitope expression was observed after incubation with AQP4‐IgG seropositive sera (IL‐17: *p* < 0.0028, IL‐6: *p* < 0.0046) compared to HC. IL‐10 pre‐treated astrocytes exhibited a trend to increased C3d epitope expression after incubation with AQP4‐IgG seropositive serum, but differences were not significant. In ROC analysis, astrocytic C3d epitope expression showed the highest AUC values (experimental and repetition level classification) after incubation with AQP4/MOG‐IgG double seronegative sera under IL‐10 and IL‐17A pre‐treatment conditions (0.75/0.73 for IL‐10 and 0.67/0.70 for IL‐17A). After exposure to AQP4‐IgG seropositive under IL‐17A pre‐treatment conditions, the AUC values were 0.62/0.61 (Figure 5B). We have included representative microscopy images of C3d immunofluorescence staining in the supplement (Figure S8A), highlighting differential expression patterns across the NMOSD subgroups. These images further illustrate the distinct astrocytic reactivity linked to AQP4‐IgG seropositive NMOSD versus MOG‐IgG seropositive or double seronegative cases, particularly in the cytokine‐pre‐treated conditions. To rule out a direct link between serum concentrations and the observed staining pattern, we performed an ELISA analysis of C3d levels in the different sera (Figure S8B). The results showed varied C3d expression among NMOSD subgroups, with only MOG‐IgG seropositive NMOSD exhibiting significantly higher C3d levels. In contrast, C3d levels in AQP4‐IgG seropositive and double seronegative NMOSD sera were not significantly elevated compared to healthy control (HC) sera. When examining potential sex‐specific differences in C3d immunofluorescence in our assay, we did not observe any distinct patterns or signals (Figure S9).

Taken together, C3d epitope expression on astrocytes showed the most significant results for AQP4‐IgG seropositive sera with an increased expression of the C3d epitope on astrocytes after IL‐17A or IL‐6 pre‐treatment, while no modulation was seen in the untreated condition (which is clearly different from the AQP4 epitope expression IF analysis).

### 3.5 AQP4 and C3d Epitope Expression Differentiates Seronegative From Seropositive NMOSD

To comprehensively analyze the distinct variations in AQP4 or C3d epitope expression among different NMOSD subgroups, we normalized each patient with NMOSD with a control participant of the same age and sex. Subsequently, we compared these normalized measurements across the different subgroups.

**FIGURE 6:**
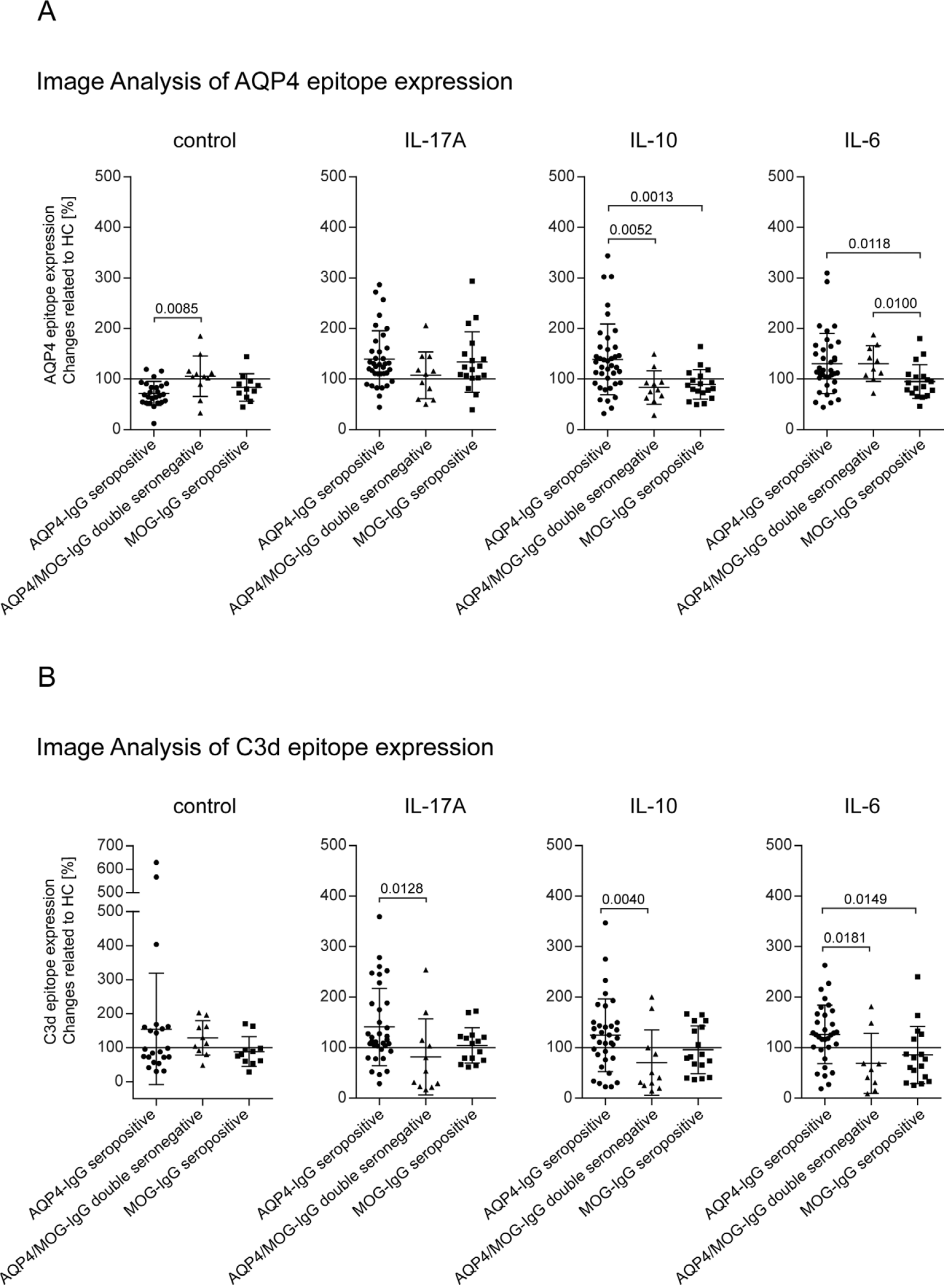
Comparison of changes in (A) AQP4 and (B) C3 epitope expression area related to HC for the different NMOSD types in astrocytes pre‐incubated with IL‐10, IL‐17, IL‐6, and control. Single values of every experiment and statistical evaluation are depicted for NMOSD‐AQP4‐IgG seropositive (*n* = 22–36), for NMOSD‐MOG/AQP4‐IgG double seronegative (*n* = 10–11), and for NMOSD‐AQP4/MOG‐IgG seropositive (*n* = 11–18). Statistical test: Mann–Whitney *U* test, *p*‐values are presented with significance determined at thresholds of *p* < 0.05, *p* < 0.01, *p* < 0.001, and *p* < 0.0001.

We found a significant reduction in AQP4 expression in astrocytes exposed to AQP4‐IgG seropositive sera under control conditions, as opposed to those treated with AQP4/MOG‐IgG double seronegative sera (*p* = 0.0085) (Figure 6A). After IL‐17A pre‐treatment, we observed a trend towards increased AQP4 expression for AQP4‐IgG seropositive and MOG‐IgG seropositive sera compared to AQP4/MOG‐IgG double seronegative sera. In an IL‐10 environment, astrocytes treated with AQP4‐IgG seropositive sera exhibited higher AQP4 expression compared to astrocytes exposed to AQP4/MOG‐IgG double seronegative sera (*p* = 0.0052) or MOG‐IgG seropositive sera (*p* = 0.0013). Upon pre‐incubating astrocytes with IL‐6, increased AQP4 expression was observed for AQP4‐IgG seropositive sera (*p* = 0.0118) and AQP4/MOG‐IgG double seronegative sera (*p* = 0.01) compared to MOG‐IgG seropositive sera.

For C3d epitope expression, in the control condition, we observed a trend to C3d epitope increase in AQP4‐IgG seropositive and AQP4/MOG‐IgG double seronegative compared to MOG‐IgG seropositive (Figure 6B). Pre‐treatment with IL‐17A led to increased C3d epitope expression in the AQP4‐IgG seropositive group compared to the AQP4/MOG‐IgG double seronegative group (*p* = 0.0128). Similarly, IL‐10 or IL‐6 resulted in increased C3d epitope expression for the NMOSD‐AQP4‐IgG seropositive group compared to the AQP4/MOG‐IgG double seronegative group (IL‐10: *p* = 0.004; IL‐6: *p* = 0.0181). In IL‐6 pre‐conditioning, C3d epitope expression was also significantly higher in AQP4‐IgG seropositive compared to MOG‐IgG seropositive sera incubated astrocytes (*p* = 0.0149).

Taken together, there are differential and multidimensional effects identifiable depending on NMOSD group and pre‐treatment condition.

### 3.6 Unbiased Analysis of AQP4 and C3d Epitopes in Control and Cytokine Pre‐Treated Astrocytes Revealed NMOSD Categorization

Since we observed distinct AQP4 and C3d epitope expression pattern of astrocytes in both the different pre‐treatment groups and the different subgroups of NMOSD, we evaluated both markers and the different pre‐treatments in a multidimensional analysis. Our NMOSD cohort was initially subdivided according to routine AQP4‐IgG and MOG‐IgG testing for comparative analysis. We were now interested in how far an unbiased analysis of this cohort using the data of our multi‐dimensional assay would categorize these patients. We performed therefore a Uniform Manifold Approximation and Projection for Dimension Reduction (UMAP) analysis based on the normalized parameters for AQP4 and C3d epitope expression, which visualizes and categorizes similar individual responses (Becht et al. 2018).

**FIGURE 7:**
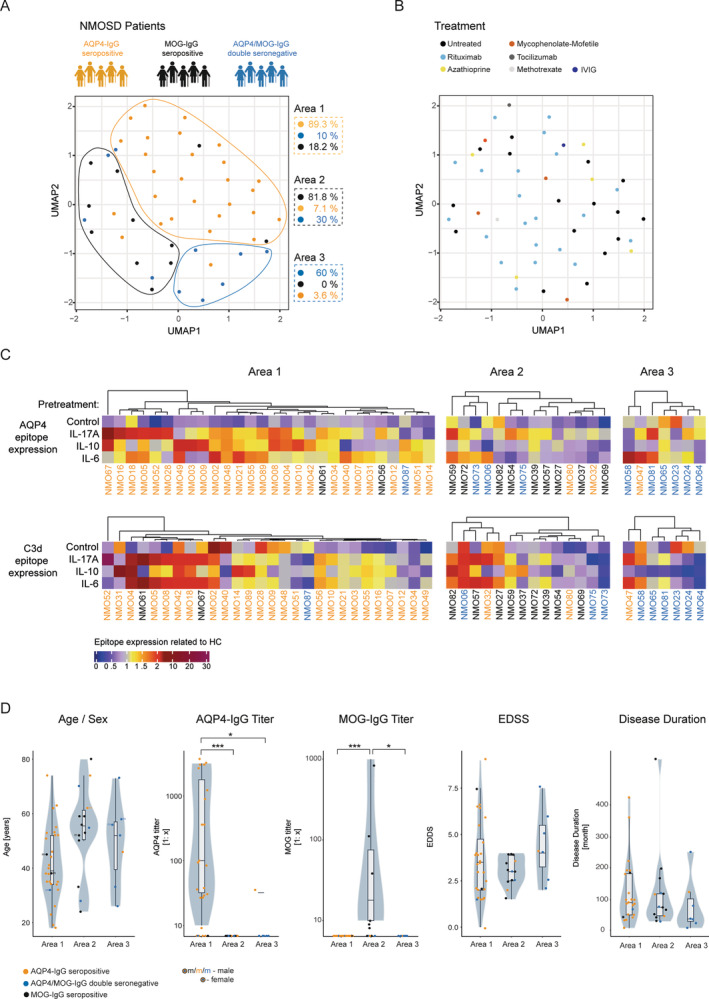
Visualization of AQP4 and C3d expression data in astrocytes after cytokine pre‐treatment with IL‐10, IL‐17A, IL‐6, or control, followed by exposure to sera from different NMOSD types, shown as (A) NMOSD subtypes and (B) treatment groups represented in UMAP (*n* = 52). (B) Regions derived from this analysis highlight differences in AQP4 and C3d expression. (C) Individual patient data and (D) associated clinical data are displayed for each region in violin plots (both *n* = 52). Statistical analysis: Kruskal–Wallis test, followed by Dunn's post hoc test. *p*‐values are indicated, with significance levels denoted as follows: *p < 0.05, **p < 0.01, and ***p < 0.001.

UMAP analysis of the data resulted in three areas of segregation, each largely corresponding AQP4‐IgG/MOG‐IgG status. In the first area, 89.3% were AQP4‐IgG seropositive; in the second area, 81.8% MOG‐IgG seropositive; and in the third area, 60% were AQP4/MOG‐IgG double seronegative (Figure 7A). The different treatment groups do not cluster in distinct regions; rather, they are randomly scattered across the entire UMAP, indicating no apparent treatment effect (Figure 7B). Heatmap analysis of the different markers revealed that area 1 is characterized by reduced expression of AQP4 and C3d epitope under control conditions and predominantly increased expression of AQP4 and C3d epitope under IL‐17, IL‐10, and IL‐6 pre‐treatment conditions following incubation with patient sera compared to HC sera (Figure 7C). Area 2 is defined by a decrease in AQP4 epitope expression under IL‐10 pre‐treatment condition and partly decreased AQP4 epitope expression under IL‐6 pre‐treatment compared to HC sera. Area 3 is characterized by reduced epitope expression of AQP4 and C3d in the IL‐10 pre‐treatment condition compared to HC serum. Looking at the demographic and clinical data of the patients revealed that the proportion of male patients was highest in area 3 (29% vs. 11% in area 1 and 14% in area 2) (Figure 7D). On average, patients in area 1 were 42 years old, followed by area 3 (49 years), area 2 (54 years). Of note, in area 2, all patients had undetectable AQP4‐IgG levels at the time of blood sampling, including those in the AQP4‐IgG seropositive subgroup. Area 3 contained the patients most affected: On average, the associated patients had the shortest disease duration (79 months) with the highest EDSS (4.4), whereas patients' area 1 and 2 had lower EDSS scores (3.66 in area 1, 1.19 in area 2) and longer disease duration (122 months in area 1, 119 months in area 2). We did not find significant correlations between disability status and response in the assays (Figure S10).

## 4 Discussion

In this study, we investigated a large cohort of patients with neuromyelitis optica spectrum disorders (NMOSD), pre‐classified based on serum IgG autoantibodies against AQP4 and MOG. Using our novel assay—based on the hypothesis that inflammatory cytokines, in conjunction with AQP4 and MOG‐IgG, are primary drivers of disease activity—we employed human astrocytes, the original target cells in AQP4‐IgG seropositive NMOSD. These astrocytes were pre‐treated with pro‐ or anti‐inflammatory cytokines and then incubated with serum from either patients or healthy controls. Pre‐treatment with specific cytokines, particularly IL‐17A, IL‐10, or IL‐6, enabled us to distinguish AQP4‐IgG seropositive sera from healthy control sera based on AQP4 and C3d epitope expression. Further experiments with a larger set of AQP4‐IgG seropositive patients confirmed the assay's effectiveness, with AQP4 and C3d epitopes demonstrating the highest diagnostic value. Unbiased UMAP analysis revealed distinct clustering of NMOSD patients according to AQP4‐IgG/MOG‐IgG status and cytokine responses, providing a more nuanced classification of patients. This approach may help identify individuals whose disease category is not fully captured by standard diagnostic tests, potentially improving detection of cases with similar biological profiles.

In recent years, the diagnosis of NMOSD has been much facilitated and our understanding of the pathophysiology of NMOSD has improved following the discovery of autoantibodies targeting AQP4 and MOG. Nevertheless, lingering questions persist, particularly concerning patients who exhibit syndromic similarities but do not test positive for either antibody. The term “seronegative NMOSD” is likely inclusive of a diverse cohort, encompassing those genuinely lacking MOG‐IgG or AQP4‐IgG, as well as individuals with false‐negative results for MOG‐IgG/AQP4‐IgG due to various reasons. This distinction is crucial, as erroneously labeling cases as seronegative NMOSD can have significant implications for both diagnosis and treatment (Wu et al. 2023). To differentiate seronegative NMOSD from AQP4‐IgG seropositive NMOSD, researchers have explored alternative biomarkers, identifying serum GFAP (but not s‐NF‐L) as a potential distinguishing marker (Carta et al. 2024). The recognition of the relevance of GFAP in NMOSD—an astrocyte‐reactivity marker—aligns with our focus on astrocytes in laboratory testing. Moreover, this study reveals heterogeneity in terms of antibody‐positive and ‐negative NMOSD cases, underscoring the inadequacy of conventional laboratory testing in capturing the complexity of the disease. This raises the question of what seronegative NMOSD truly signifies—whether patients consistently lack AQP4‐IgG or MOG‐IgG, or if the timing of antibody testing is insufficient for a reliable result. Previous research confirms that seroconversion to negative autoantibody testing is a relevant factor in MOGAD, adding another layer to the understanding of seronegative NMOSD (Forcadela et al. 2024). Another factor that might influence the outcome of testing is what type of specimen is used for autoantibody testing. Recent reports confirm that there are some cases where antibody testing for MOG‐IgG (but not for AQP4‐IgG) is positive only in the CSF but not in the serum, probably owing to low serum titers and limited sensitivity of the currently used assays. While AQP4‐IgG in CSF seems well correlated with serum titers (Takahashi et al. 2007), recent reports validated the increased sensitivity of MOG‐IgG antibody testing, when cerebrospinal fluid (CSF) is assessed in addition (Matsumoto et al. 2023). Similarly, single reports exist on patients positive for AQP4‐IgG only in the CSF. Finally, some patients with “seronegative NMOSD” have been reported to respond to antibody‐ or B cell‐depleting therapies. All these findings support the hypothesis that some patients classified as double‐seronegative may, in fact, be false‐negatives. Tests assessing the effects of antibody binding rather than the antibody itself, such as the assay used in this study, could help to improve the detection of genuinely antibody‐associated NMOSD cases.

Specific binding of autoantibodies to astrocytes may have different outcomes. The most far‐reaching is cytotoxicity. However, recent data show that this is not the only relevant mechanism involved in the pathogenesis of NMOSD. NMO‐IgG has been shown to modulate AQP4 and EAAT‐2 expression via astrocytic FcγR‐mediated internalization of the AQP4/NMO‐IgG complex as well as astrocytic IL‐6 expression (Hinson et al. 2017). These additional effects of AQP4‐IgG binding to astrocytes may explain the heterogeneity of disease manifestations among patients with NMOSD. There might be autoantibodies that do not bind directly to AQP4 but may target molecules relevant for the correct positioning or functioning of AQP4, which might be identifiable with our assay, but not in conventional live cell assays using non‐astrocytic immortalized cells. This has been shown for myasthenia gravis (MG), where seronegative cases were identified with similar alterations of the motor end plate as typically found in seropositive acetylcholine receptor positive MG (Nagaoka et al. 2022). In our assay, we have developed a multidimensional setting that incorporates not only cell lysis but also other inflammation‐induced alterations of astrocyte biology. Further, astrocytes closely interact with microglia—a potent source of IL‐6—which have been shown to be activated by astrocyte‐produced C3a (Chen et al. 2020). This is in line with our data showing that astrocytic C3d epitope is upregulated upon IL‐6 pre‐treatment (in the NMOSD‐AQP4‐IgG seropositive group), which supports a strong interactivity between astrocyte and microglia reactivity. However, it is still unclear whether the C3d epitope detected in the C3 complex or as free cleaved C3d is produced by the astrocytes or whether it also partially comes from the serum.

Other factors, i.e., cytokines or complement factors, may equally, even more effectively or complimentarily, identify the underlying disease pathology. Insight into this possibility comes from the observation of highly effective treatment responses to inhibiting C5 (Pittock et al. 2019) or neutralizing IL‐6 (Yamamura et al. 2019) in AQP4‐IgG seropositive NMOSD, which highlight the general significance of some of these factors. In an animal model, a recent study showed that IL‐6 and IL‐17A are relevant factors for disease pathology and are counter‐acted by interferon‐gamma (IFNg) (Arellano et al. 2024), most likely by influencing Th17 vs. Th1 effector phenotypes. The relevance of IL‐6 and IL‐1b have been supported by data that show that human AQP4‐IgG1 induce these cytokines in ex vivo brain microvessel preparations (Cobo‐Calvo et al. 2020). In this study, comprehensive effects were observed for molecules relevant for the integrity of the blood–brain barrier (BBB) (Takeshita et al. 2021). The hypothesis that cellular infiltrates contribute critically to the pathophysiology also stems from the observation that targeting the cellular elements with immunosuppressive drugs is efficient in treating NMOSD (Cree et al. 2019; Tahara et al. 2020). Our data show that cytokine treatment of astrocytes crucially alters their response to autoantibody binding. While the control condition without cytokine pre‐treatment led to downregulation of AQP4 expression area, in particular IL‐6 and IL‐17A pre‐treatment increased AQP4 expression area after incubation with NMOSD‐AQP4‐IgG seropositive sera. When simultaneous changes in cell morphology are observed, it appears that increased expression of AQP4 is not responsible, but that the cells themselves undergo changes in structure and surface properties. In this context, effects of co‐culturing iPSC‐derived astrocytes with iPSC‐derived monocytes/microglia in context of inflammatory cytokines and incubated with patient serum would indeed add another dimension of cellular immunity which may reflect even better the in vivo situation.

Some limitations of our study deserve careful consideration. The rarity of the disease implies a limited number of patients included in the study. Additionally, there are notable sex differences in the prevalence of NMOSD subtypes, with MOG‐IgG seropositive NMOSD showing a lower female preponderance, while AQP4‐IgG seropositive NMOSD has a striking 9:1 female‐to‐male ratio (Arnett et al. 2024; Uzawa et al. 2024). While we recognize that our study is underpowered for a robust analysis of sex‐specific effects due to the limited number of male NMOSD patients, our current data do not reveal any substantial or consistent trends (with a possible exception for MOG‐IgG positive NMOSD sera in IL‐6 pre‐conditioned astrocytes) that would indicate sex‐based differences. We acknowledge that a larger cohort of male NMOSD patients would be necessary to draw more definitive conclusions regarding potential sex‐specific variations in disease presentation or biomarker expression.

The retrospective design coupled with variations in the time points at which sera were obtained in relation to disease activity and treatment may impact the accuracy of test results. Concerning the test conditions: While serum‐containing media are commonly employed in cell culture, including astrocyte cultures(McCarthy and de Vellis 1980), certain considerations and potential issues should be acknowledged. Previous studies have elucidated an impact of fetal bovine serum (FBS) on astrocytic phenotype (Zhang et al. 2016). This circumstance poses a general caveat regarding reports on astrocyte activation or “reactivity.” Under our standard culture conditions, where the astrocyte differentiation medium includes 1% FBS, only a minority of astrocytes exhibited positivity for C3, a marker of reactive (A1) astrocytes (Liddelow and Barres 2017). However, the use of human sera (10%) holds the potential to induce autoantibody‐independent effects, leading to astrocyte reactivity that is neither disease‐dependent nor specific. In general, the human astrocytes used in this study are not primary cultures or slice cultures of astrocytes, which might behave differently in situ or under physiological conditions. To address the potential of serum itself inducing astrocytic changes independently of autoantibody specificity, we included healthy control (HC) serum as a baseline in our assays. Any significant shifts in AQP4 and C3d expression relative to HC serum exposure were primarily observed in AQP4‐IgG seropositive NMOSD samples, suggesting an autoantibody‐specific effect. Additionally, no significant upregulation was seen in astrocytes treated with MOG‐IgG seropositive serum (which were highest in expression of C3d epitope) in the absence of specific pre‐treatment, further supporting target cell‐specific autoantibody dependency.

It is possible that C3d seen in IF staining originates both from serum transfer and from astrocytic production under cytokine influence. As an experimental limitation, distinguishing between these two sources could be achieved with targeted C3 mRNA knockdown or by applying purified C3/C3d‐deficient serum, if feasible in future studies.

In this pilot study, we analyzed a singular serum sample per patient and samples were collected at varying time points in the patients' disease course. This may have caused false‐negative results in individual cases, particularly if samples were taken during stable disease and/or under long‐term treatments. Some patients may be antibody‐positive only in the CSF but not serum. Consequently, focusing on samples from untreated patients, samples taken at disease onset or during subsequent relapses, follow‐up samples taken during different disease stages, and including also CSF samples could further improve the sensitivity and clinical utility of our assay and provide further valuable insights into the type and extent of astrocytopathy in patients with NMOSD.

## 5 Author Contributions

M.A. and V.S. designed the research project and the experiments. M.A. and F.F. conducted the experiments. M.A. and B.M. analyzed and evaluated the data. D.Z. provided support in the statistical analysis of the data. P.S., A.K., C.O., K.R., T.S.‐H., F.P., and V.S. were involved in patient care and the collection of serum samples for the experiments, as well as overseeing the patient cohort. S.J. characterized the serum samples and provided corrections to the manuscript. M.A. and V.S. wrote the manuscript. F.P. served as a consultant for the project and reviewed the final manuscript.

## Ethics Statement

The study was approved by the institutional ethics committee of the Charité—Universitäts‐medizin Berlin (EA1/362/20) and follows the guidelines of the Declaration of Helsinki for the conduct of the study. The participants provided their written informed consent to participate in this study.

## Conflicts of Interest

S.J. reports no conflicts of interest. V.S. has received unrelated research grants from Novartis Pharma and Alexion. V.S. and F.P. have received funding for this study from Roche.

## Permission to Reproduce Materials From Other Sources and Clinical Trail Registration

No materials have been reproduced from other sources. The study is not a clinical trial.

## Supporting information


**Figure S1.** Overview of sera used in the study. (A) Sera were collected from 96 women (42 healthy controls and 54 NMOSD patients) and 19 men (6 healthy controls and 13 NMOSD patients). A violin plot illustrates the age distribution across subgroups. (B) Sera from 38 NMOSD patients tested positive for AQP4‐IgG, 11 tested negative for both AQP4‐IgG and MOG‐IgG, and 18 tested negative for AQP4‐IgG but positive for MOG‐IgG. Sera from a total of 48 healthy individuals, matched for age (±7 years) and gender to each patient, were selected as controls.

**Figure S2.** Analysis of AQP4 epitope expression area per nucleus in astrocytes incubated with varying cytokine concentrations and NMOSD‐AQP4‐IgG seropositive or HC serum. Astrocytes were pre‐incubated with (A) TNFα, (B) IL‐1β, (C) IL‐6, (D) IL‐10, and (E) IL‐17A at concentrations of 0, 0.1, 1, 10, and 50 ng/mL. After 24 h, cells were exposed to 10% HC serum or NMOSD serum. Image analysis was performed using ImageJ (*n* = 5, Mann–Whitney test, *p* < 0.05).

**Figure S3.** Analysis of EAAT2 epitope expression area per nucleus in astrocytes incubated with varying cytokine concentrations and either NMOSD‐AQP4‐IgG seropositive or HC serum. Astrocytes were pre‐incubated with (A) TNFα, (B) IL‐1β, (C) IL‐6, (D) IL‐10, and (E) IL‐17, each at concentrations of 0, 0.1, 1, 10, and 50 ng/mL. After 24 h, cells were exposed to 10% HC serum or NMOSD serum. Image analysis was performed using ImageJ (*n* = 5, Mann–Whitney test, *p* < 0.05).

**Figure S4.** Assessment of astrocyte cell viability in response to varying cytokine concentrations and NMOSD‐AQP4‐IgG seropositve or HC serum treatment. Astrocytes were pre‐incubated with (A) TNFα, (B) IL‐1β, (C) IL‐6, (D) IL‐10, and (E) IL‐17 at concentrations of 0, 0.1, 1, 10, and 50 ng/mL. After 24 h, cells were exposed to 10% HC serum (baseline set to 100%) or NMOSD serum. Data represent *n* = 5, Mann–Whitney test, *p* < 0.05.

**Figure S5.** AQP4 epitope expression in astrocytes pre‐incubated with 10 ng/mL IL‐17A, IL‐10, or 1 ng/mL IL‐6, followed by exposure to 10% serum from AQP4‐IgG seropositive, MOG/AQP4‐IgG double seronegative, and MOG‐IgG seropositive patients. Representative images are provided.

**Figure S6.** Analysis of epitope expression of AQP4 in astrocytes after pre‐incubated with 10 ng/ mL IL‐17A, IL‐10 or 1 ng/mL IL‐6 and exposure to 10% sera from AQP4‐IgG seropositive, MOG/AQP4‐IgG double seronegative and for AQP4‐MOG‐IgG seropositive patients (each *n* = 5–7) regarding process count, process area, and total area. Statistical test: Kruskal–Wallis test, followed by Dunn’s post hoc test, *p*‐values are indicated, with significance levels denoted as follows: *p < 0.05.

**Figure S7.** Changes in AQP4 expression area relative to corresponding HC for each NMOSD subtype, analyzed by patient sex in astrocytes pre‐incubated with IL‐10, IL‐17A, IL‐6, or control. Individual values and statistical evaluations are shown for AQP4‐IgG seropositive patients (females, *n* = 20–36; males, *n* = 1–3), double seronegative MOG/AQP4‐IgG patients (females, *n* = 7; males, *n* = 3–4), and MOG‐IgG seropositive patients (females, *n* = 7; males, *n* = 3–5). Statistical test: Mann–Whitney *U* test, with *p*‐values indicated; significance levels are denoted as follows: * *p* < 0.05 and ** *p* < 0.01.

**Figure S8.** Detection of C3d in astrocytes and sera from NMOSD patients. (A) C3d epitope expression in astrocytes pre‐incubated with 10 ng/mL IL‐17A, IL‐10, or 1 ng/mL IL‐6, followed by exposure to 10% serum from AQP4‐IgG seropositive, MOG/AQP4‐IgG double seronegative, and MOG‐IgG seropositive patients. Representative images are provided. (B) C3d concentrations in sera from AQP4‐IgG seropositive (*n* = 12), MOG/AQP4‐IgG double seronegative (*n* = 8), MOG‐IgG seropositive (*n* = 11), and HC individuals (*n* = 10), as detected by ELISA. Statistical test: Mann–Whitney U test; *p*‐values are indicated with significance levels denoted as follows: * *p* < 0.05 and ** *p* < 0.01.

**Figure S9.** Changes in C3d expression area relative to corresponding HC for each NMOSD subtype, analyzed by patient sex in astrocytes pre‐incubated with IL‐10, IL‐17A, IL‐6, or control. Individual values and statistical evaluations are shown for AQP4‐IgG seropositive patients (females, *n* = 20–36; males, *n* = 1–3), double seronegative MOG/AQP4‐IgG patients (females, *n* = 7; males, *n* = 3–4), and MOG‐IgG seropositive patients (females, *n* = 7; males, *n* = 3–5). Statistical test: Mann–Whitney *U* test, with *p*‐values indicated when significant: *p < 0.05 and **p < 0.01.

**Figure S10.** Correlation between (A) C3d epitope expression (B) AQP4 epitope expression in astrocytes after pre‐incubated with 10 ng/mL IL‐17A, IL‐10 or 1 ng/mL IL‐6 and exposure to 10% sera from AQP4‐IgG seropositive, MOG/AQP4‐IgG double seronegative and for AQP4‐MOG‐IgG seropositive patients and the clinical parameter EDSS of each patient (AQP4 epitope expression: control *n* = 44, IL‐17 *n* = 60, IL‐10 *n* = 63, IL‐6 *n* = 64; C3 epitope expression: control *n* = 40, IL‐17 *n* = 59, IL‐10 and IL‐6 *n* = 62). Statistical test: Spearman’s rank correlation test, no significant correlation detected.

## Data Availability

The data that support the findings of this study are available on request from the corresponding author. The data are not publicly available due to privacy or ethical restrictions.
