# Supplementary figures and images for: Distinguishing Neuromyelitis Optica Spectrum Disorders Subtypes: A Study on AQP4 and C3d Epitope Expression in Cytokine‐Primed Human Astrocytes
Source: Glia. 2025 Jan 27;73(5):1090–106. doi: 10.1002/glia.24675 (PMC11920679; doi:10.1002/glia.24675)

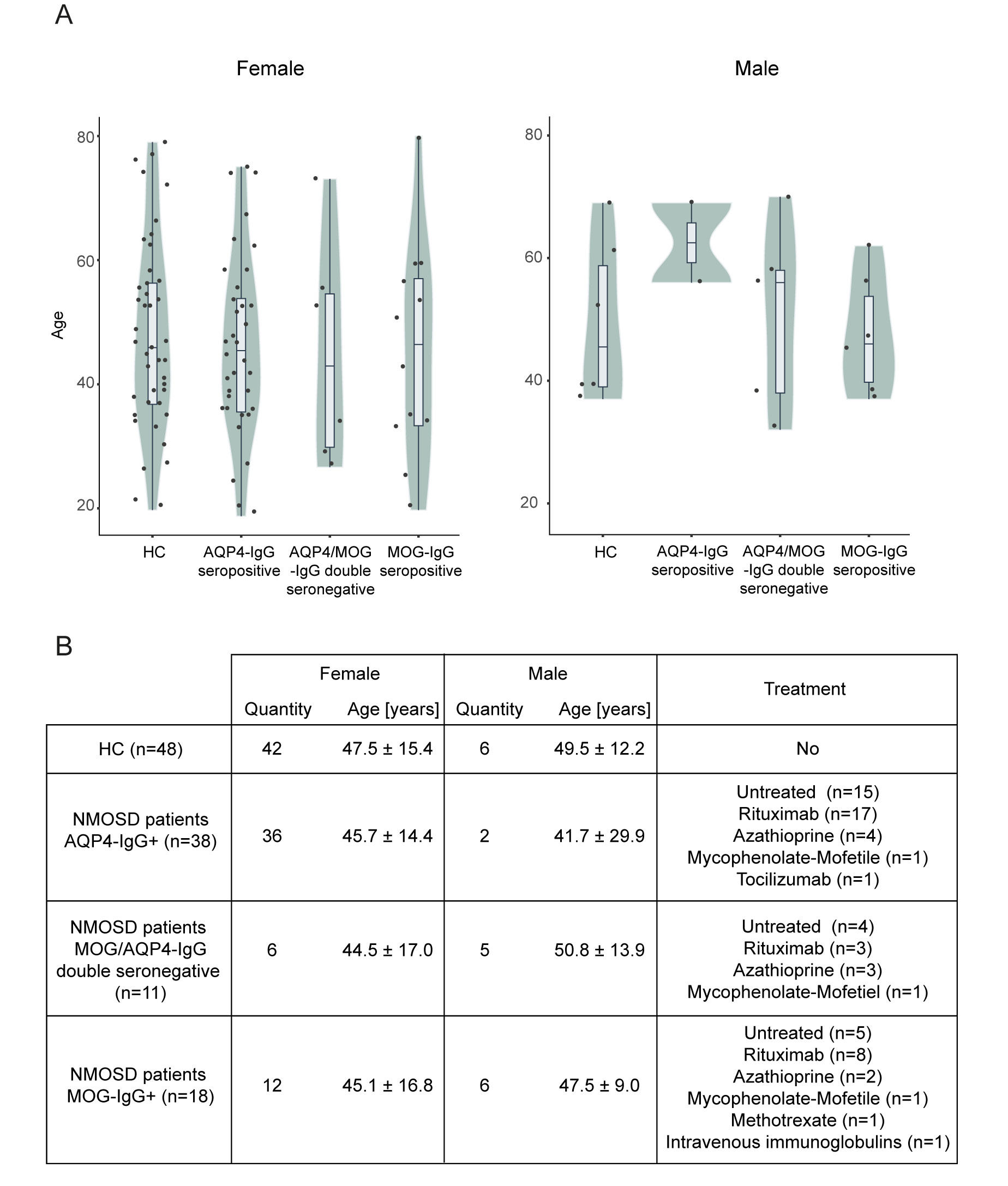

Supplement: Supplementary file 1 — Figure S1. Overview of sera used in the study. (A) Sera were collected from 96 women (42 healthy controls and 54 NMOSD patients) and 19 men (6 healthy controls and 13 NMOSD patients). A violin plot illustrates the age distribution across subgroups. (B) Sera from 38 NMOSD patients tested positive for AQP4‐IgG, 11 tested negative for both AQP4‐IgG and MOG‐IgG, and 18 tested negative for AQP4‐IgG but positive for MOG‐IgG. Sera from a total of 48 healthy individuals, matched for age (±7 years) and gender to each patient, were selected as controls. [file GLIA-73-1090-s010.tif]

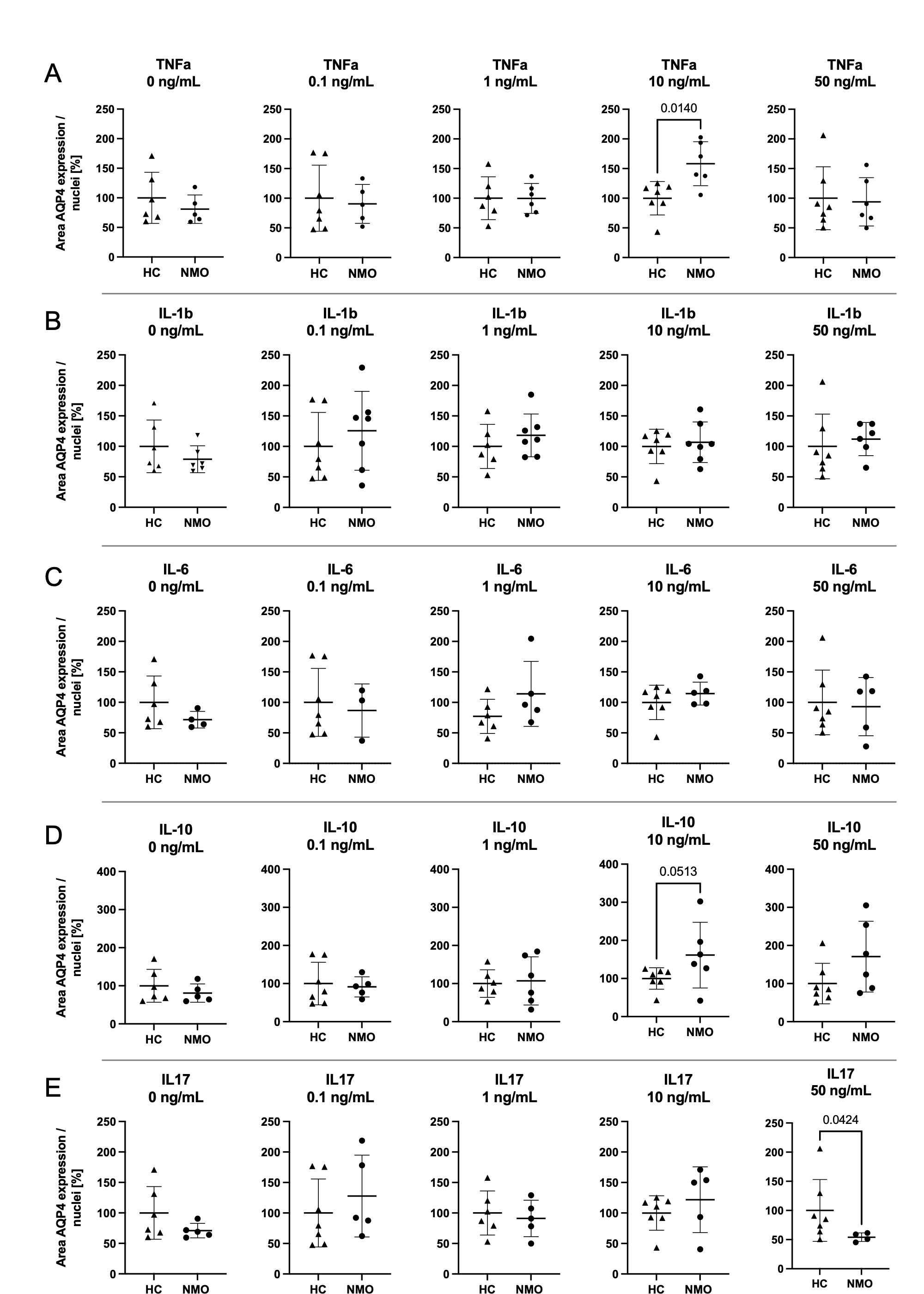

Supplement: Supplementary file 2 — Figure S2. Analysis of AQP4 epitope expression area per nucleus in astrocytes incubated with varying cytokine concentrations and NMOSD‐AQP4‐IgG seropositive or HC serum. Astrocytes were pre‐incubated with (A) TNFα, (B) IL‐1β, (C) IL‐6, (D) IL‐10, and (E) IL‐17A at concentrations of 0, 0.1, 1, 10, and 50 ng/mL. After 24 h, cells were exposed to 10% HC serum or NMOSD serum. Image analysis was performed using ImageJ (n = 5, Mann–Whitney test, p < 0.05). [file GLIA-73-1090-s002.tiff]

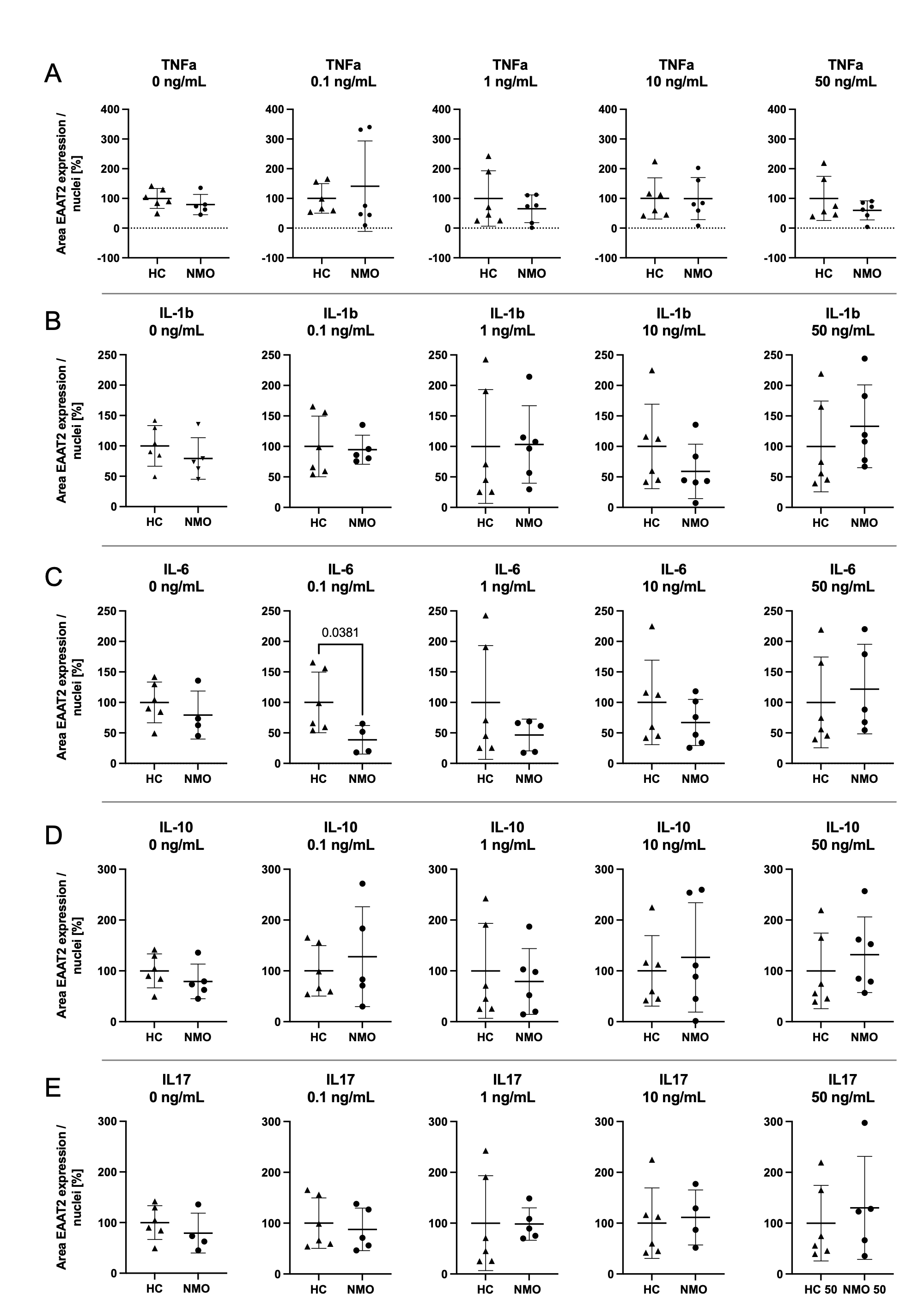

Supplement: Supplementary file 3 — Figure S3. Analysis of EAAT2 epitope expression area per nucleus in astrocytes incubated with varying cytokine concentrations and either NMOSD‐AQP4‐IgG seropositive or HC serum. Astrocytes were pre‐incubated with (A) TNFα, (B) IL‐1β, (C) IL‐6, (D) IL‐10, and (E) IL‐17, each at concentrations of 0, 0.1, 1, 10, and 50 ng/mL. After 24 h, cells were exposed to 10% HC serum or NMOSD serum. Image analysis was performed using ImageJ (n = 5, Mann–Whitney test, p < 0.05). [file GLIA-73-1090-s008.tiff]

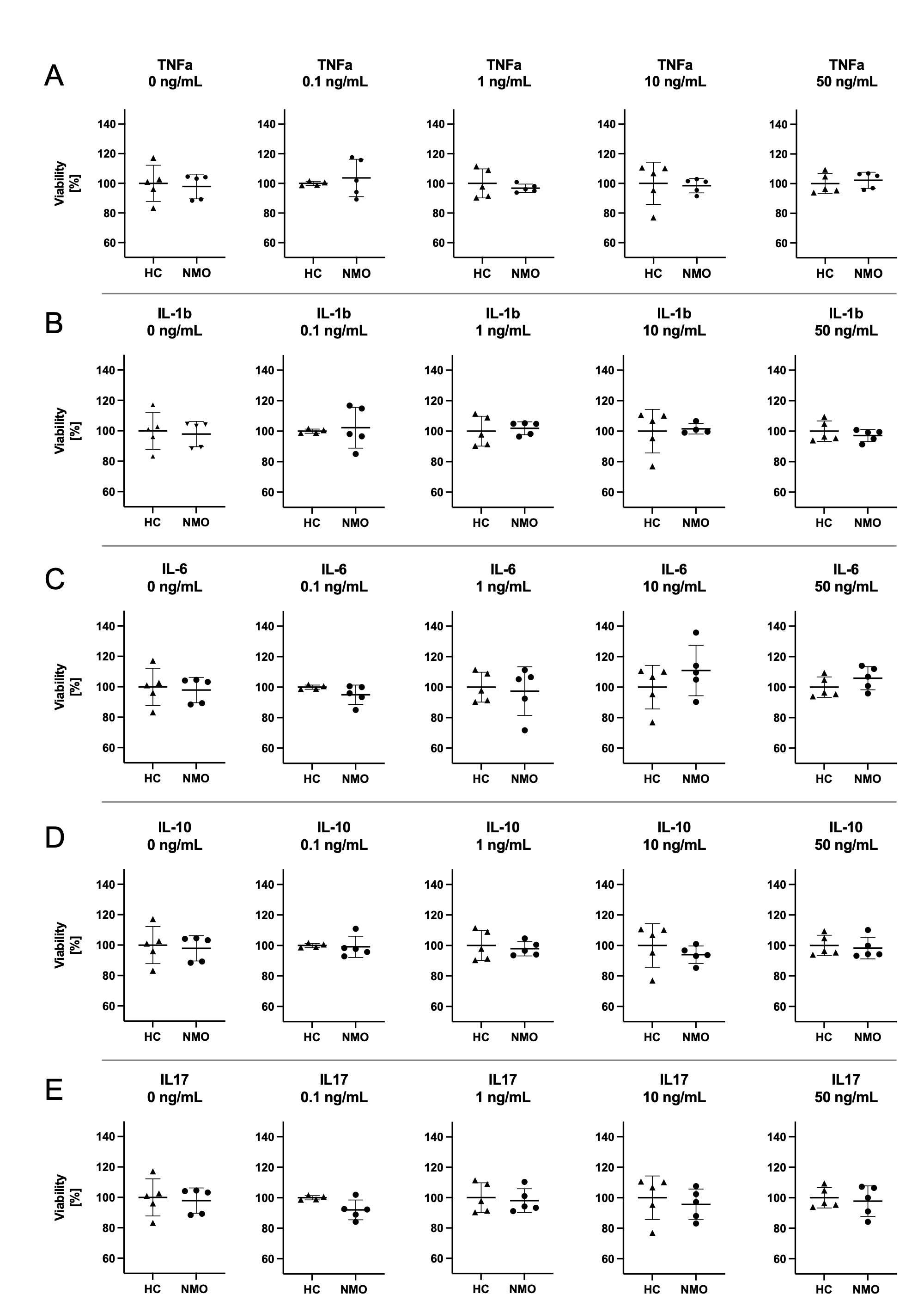

Supplement: Supplementary file 4 — Figure S4. Assessment of astrocyte cell viability in response to varying cytokine concentrations and NMOSD‐AQP4‐IgG seropositve or HC serum treatment. Astrocytes were pre‐incubated with (A) TNFα, (B) IL‐1β, (C) IL‐6, (D) IL‐10, and (E) IL‐17 at concentrations of 0, 0.1, 1, 10, and 50 ng/mL. After 24 h, cells were exposed to 10% HC serum (baseline set to 100%) or NMOSD serum. Data represent n = 5, Mann–Whitney test, p < 0.05. [file GLIA-73-1090-s006.tiff]

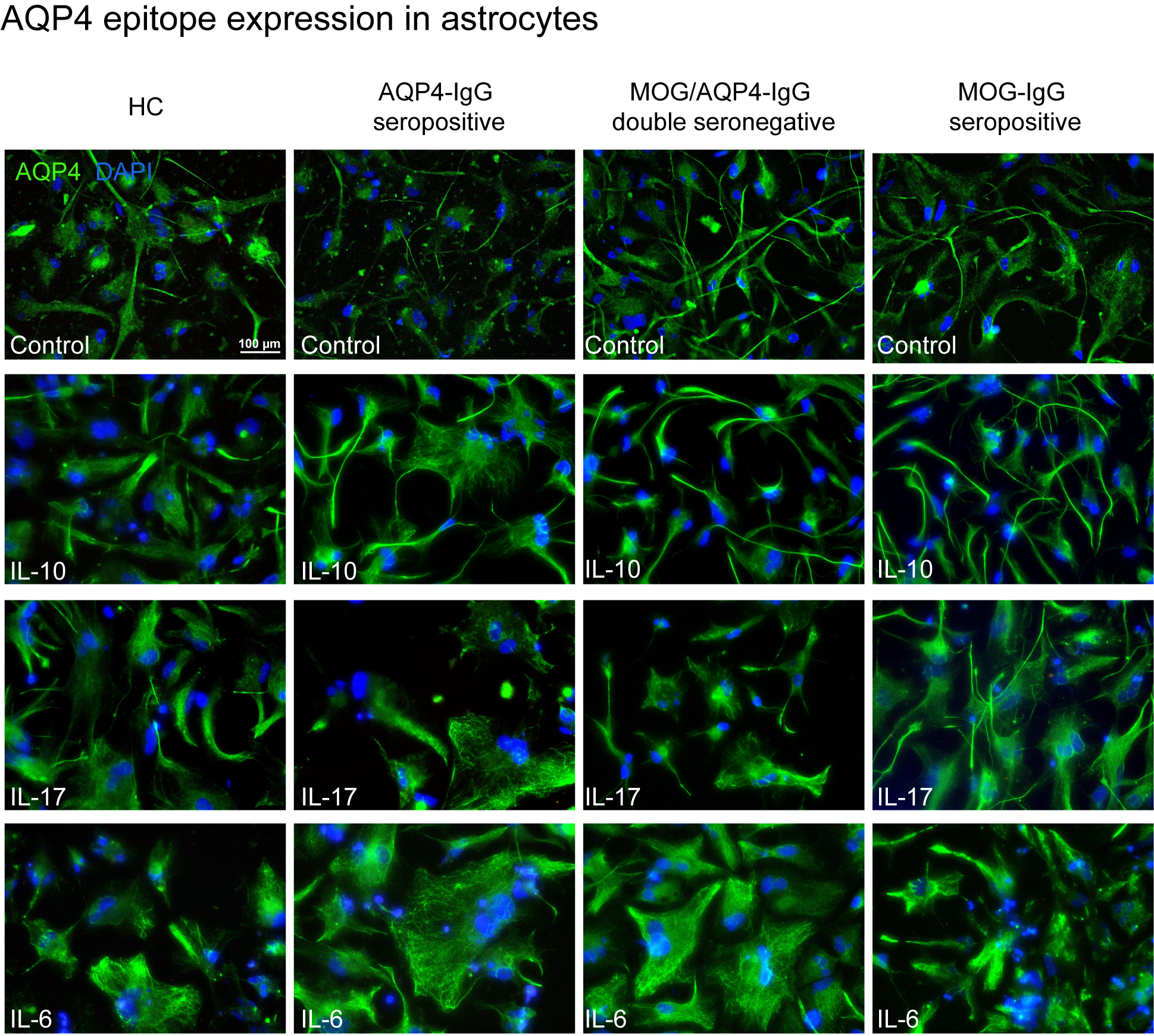

Supplement: Supplementary file 5 — Figure S5. AQP4 epitope expression in astrocytes pre‐incubated with 10 ng/mL IL‐17A, IL‐10, or 1 ng/mL IL‐6, followed by exposure to 10% serum from AQP4‐IgG seropositive, MOG/AQP4‐IgG double seronegative, and MOG‐IgG seropositive patients. Representative images are provided. [file GLIA-73-1090-s005.tif]

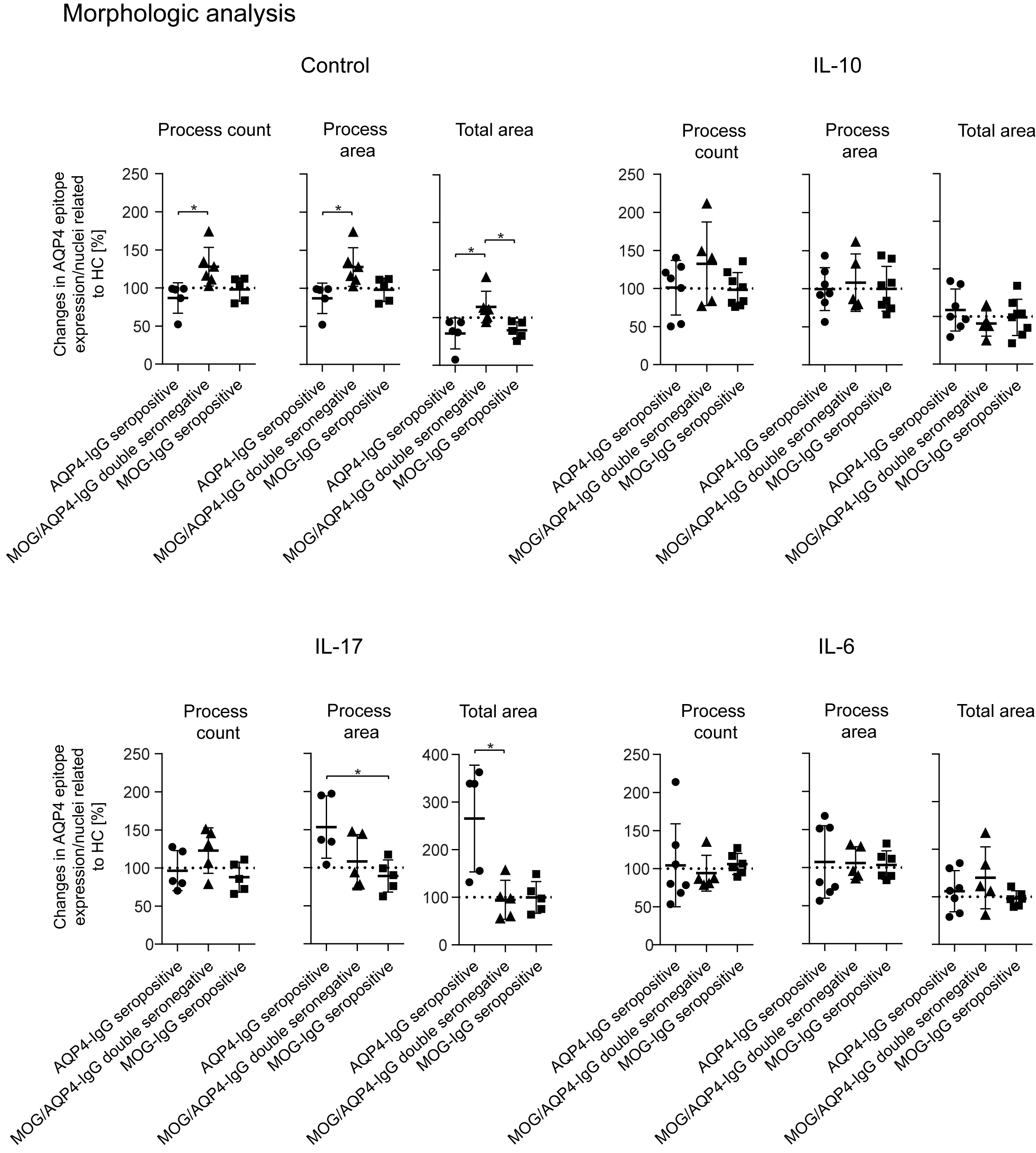

Supplement: Supplementary file 6 — Figure S6. Analysis of epitope expression of AQP4 in astrocytes after pre‐incubated with 10 ng/ mL IL‐17A, IL‐10 or 1 ng/mL IL‐6 and exposure to 10% sera from AQP4‐IgG seropositive, MOG/AQP4‐IgG double seronegative and for AQP4‐MOG‐IgG seropositive patients (each n = 5–7) regarding process count, process area, and total area. Statistical test: Kruskal–Wallis test, followed by Dunn’s post hoc test, p‐values are indicated, with significance levels denoted as follows: *p < 0.05. [file GLIA-73-1090-s007.tif]

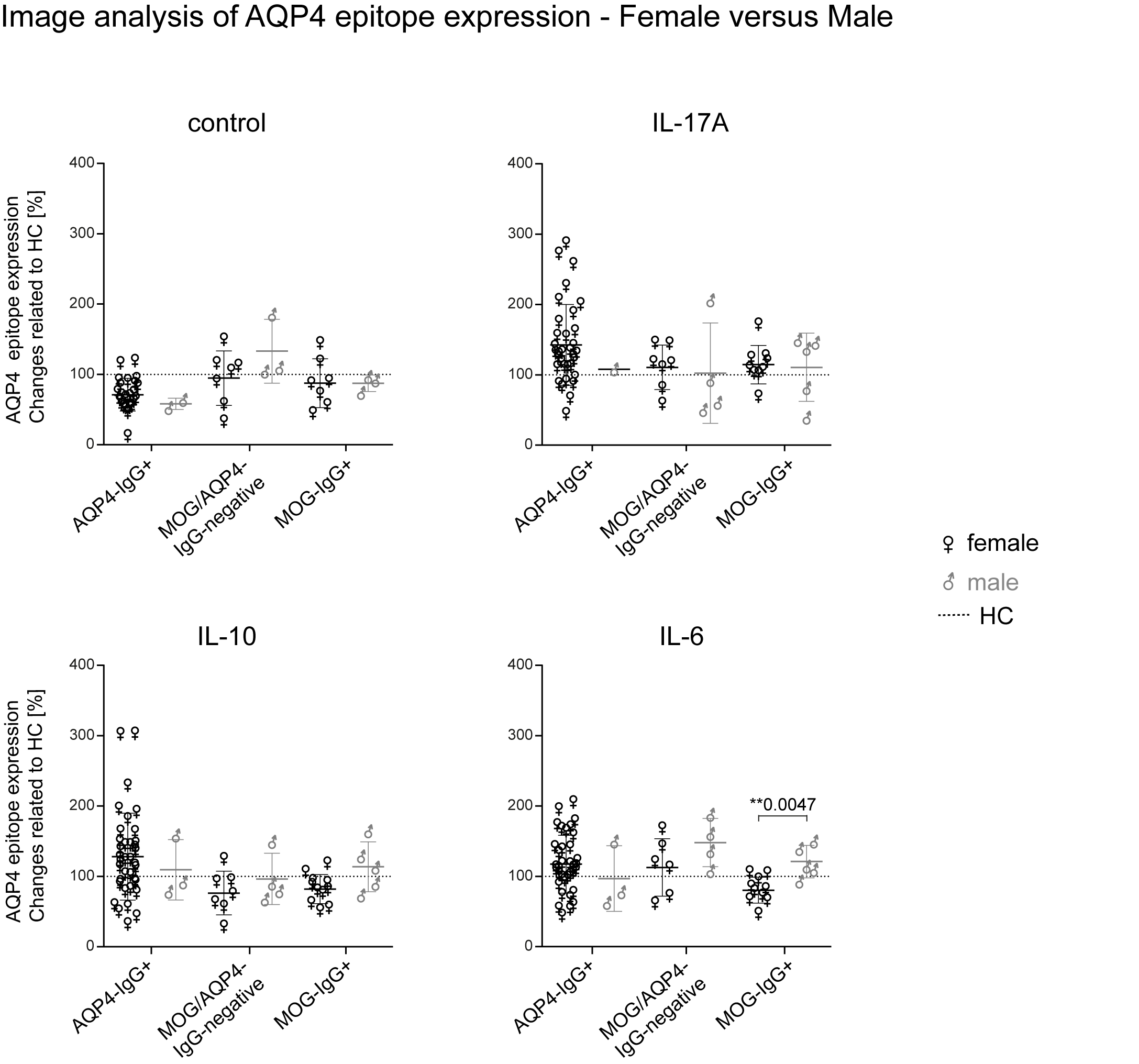

Supplement: Supplementary file 7 — Figure S7. Changes in AQP4 expression area relative to corresponding HC for each NMOSD subtype, analyzed by patient sex in astrocytes pre‐incubated with IL‐10, IL‐17A, IL‐6, or control. Individual values and statistical evaluations are shown for AQP4‐IgG seropositive patients (females, n = 20–36; males, n = 1–3), double seronegative MOG/AQP4‐IgG patients (females, n = 7; males, n = 3–4), and MOG‐IgG seropositive patients (females, n = 7; males, n = 3–5). Statistical test: Mann–Whitney U test, with p‐values indicated; significance levels are denoted as follows: * p < 0.05 and ** p < 0.01. [file GLIA-73-1090-s003.tif]

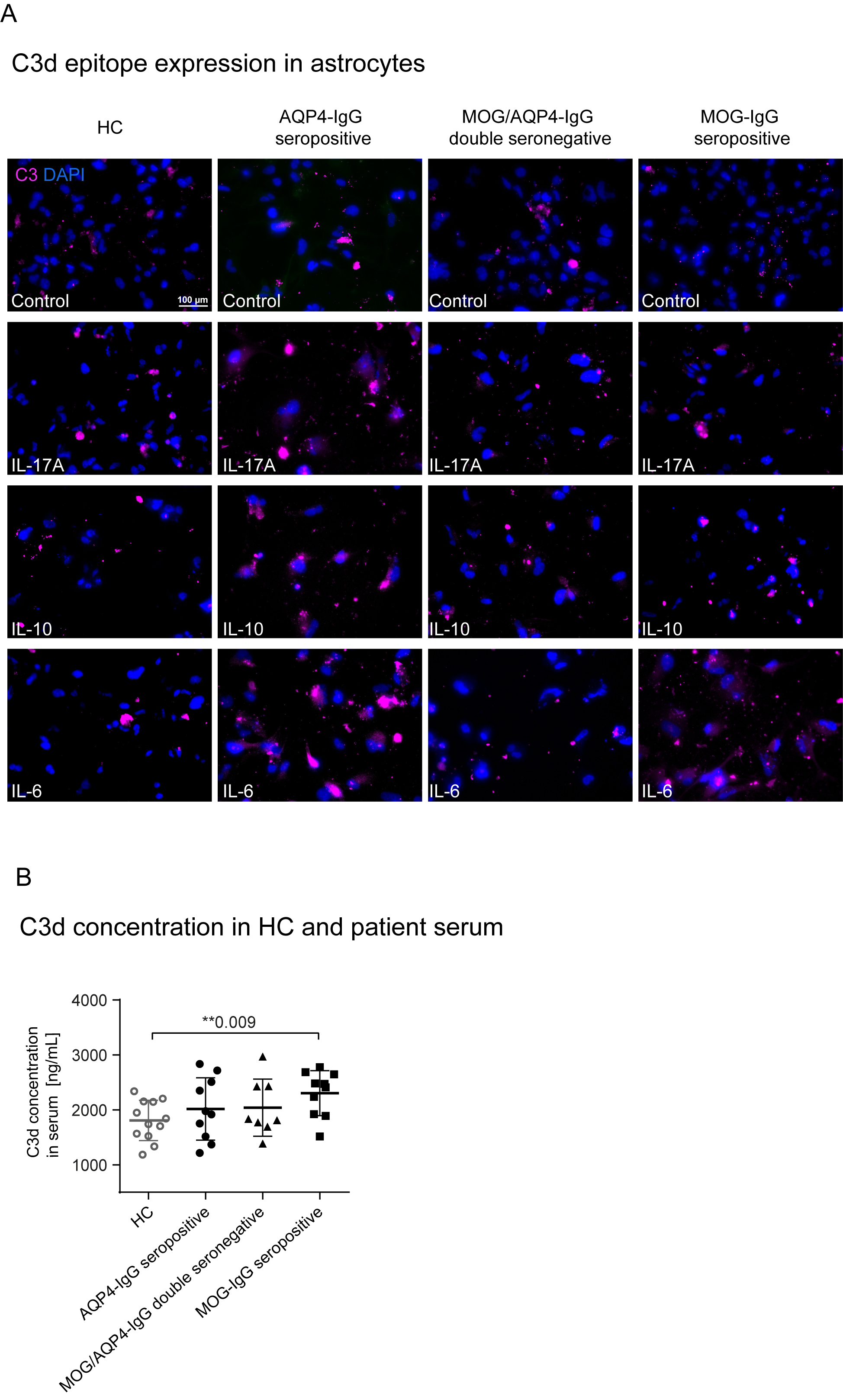

Supplement: Supplementary file 8 — Figure S8. Detection of C3d in astrocytes and sera from NMOSD patients. (A) C3d epitope expression in astrocytes pre‐incubated with 10 ng/mL IL‐17A, IL‐10, or 1 ng/mL IL‐6, followed by exposure to 10% serum from AQP4‐IgG seropositive, MOG/AQP4‐IgG double seronegative, and MOG‐IgG seropositive patients. Representative images are provided. (B) C3d concentrations in sera from AQP4‐IgG seropositive (n = 12), MOG/AQP4‐IgG double seronegative (n = 8), MOG‐IgG seropositive (n = 11), and HC individuals (n = 10), as detected by ELISA. Statistical test: Mann–Whitney U test; p‐values are indicated with significance levels denoted as follows: * p < 0.05 and ** p < 0.01. [file GLIA-73-1090-s001.tif]

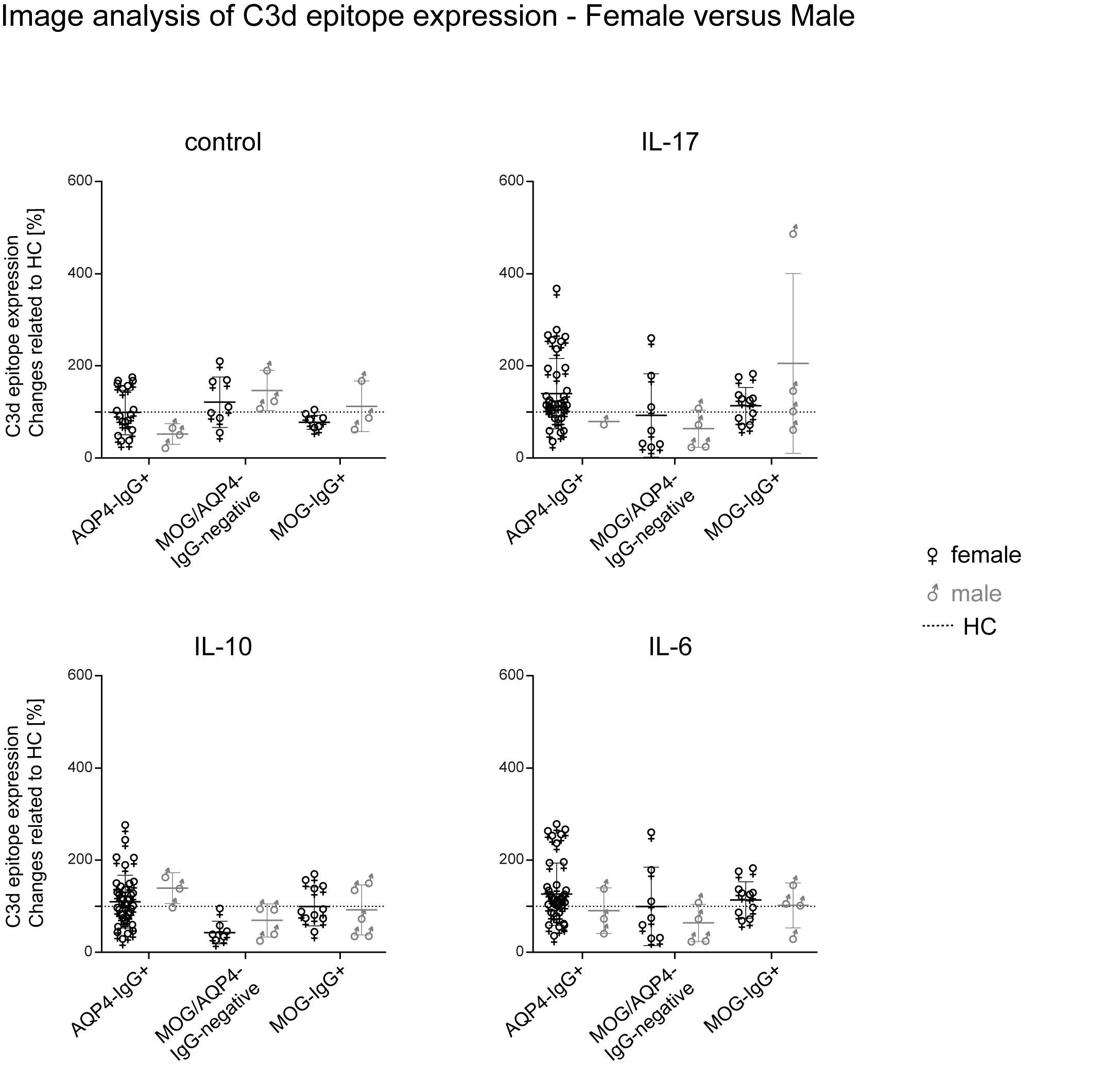

Supplement: Supplementary file 9 — Figure S9. Changes in C3d expression area relative to corresponding HC for each NMOSD subtype, analyzed by patient sex in astrocytes pre‐incubated with IL‐10, IL‐17A, IL‐6, or control. Individual values and statistical evaluations are shown for AQP4‐IgG seropositive patients (females, n = 20–36; males, n = 1–3), double seronegative MOG/AQP4‐IgG patients (females, n = 7; males, n = 3–4), and MOG‐IgG seropositive patients (females, n = 7; males, n = 3–5). Statistical test: Mann–Whitney U test, with p‐values indicated when significant: *p < 0.05 and **p < 0.01. [file GLIA-73-1090-s004.tif]

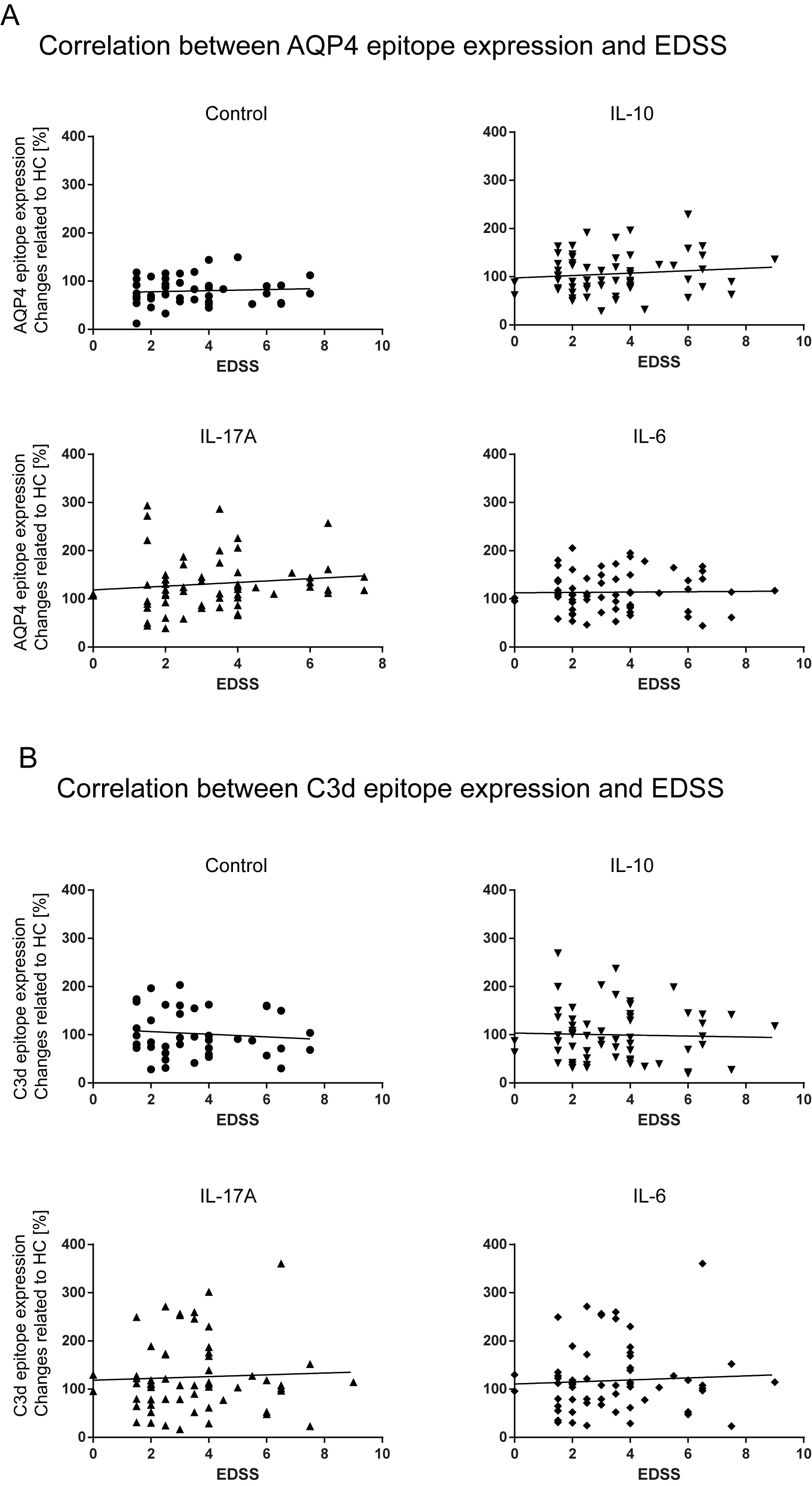

Supplement: Supplementary file 10 — Figure S10. Correlation between (A) C3d epitope expression (B) AQP4 epitope expression in astrocytes after pre‐incubated with 10 ng/mL IL‐17A, IL‐10 or 1 ng/mL IL‐6 and exposure to 10% sera from AQP4‐IgG seropositive, MOG/AQP4‐IgG double seronegative and for AQP4‐MOG‐IgG seropositive patients and the clinical parameter EDSS of each patient (AQP4 epitope expression: control n = 44, IL‐17 n = 60, IL‐10 n = 63, IL‐6 n = 64; C3 epitope expression: control n = 40, IL‐17 n = 59, IL‐10 and IL‐6 n = 62). Statistical test: Spearman’s rank correlation test, no significant correlation detected. [file GLIA-73-1090-s009.tif]
